# Interfacial Denaturation at the Droplet Simplifies the Formation of Drug‐Loaded Protein Nanocapsules to Enhance Immune Response of Cells

**DOI:** 10.1002/advs.202403668

**Published:** 2024-07-08

**Authors:** Sharafudheen Pottanam Chali, Jinhong Kang, Michael Fichter, Kai Robert Speth, Volker Mailänder, Katharina Landfester

**Affiliations:** ^1^ Max Planck Institute for Polymer Research Ackermannweg 10 55128 Mainz Germany; ^2^ Department of Dermatology University Medical Center Mainz Langenbeckstraße 1 55131 Mainz Germany

**Keywords:** denaturation, immunotherapy, miniemulsion, nanocapsules, proteins

## Abstract

Nanocapsules enable multicomponent encapsulation of therapeutic cargoes with high encapsulation content and efficiency, which is vital for cancer immunotherapy. In the past, chemical crosslinking is used to synthesize nanocapsules, which can impede the regulatory approval process. Therefore, a new class of protein nanocapsules is developed by eliminating the need for chemical crosslinking by utilizing protein denaturation through a process that is referred to as “baking at the droplet interface”. Such protein nanocapsules with antigens incorporated in the shell and a combination of encapsulated drugs showed an enhancement in the immune response of cells.

## Graphical abstract

Protein nanocapsules are synthesized utilizing miniemulsion and exploiting inherent properties of proteins, namely denaturation. This novel strategy eliminates the necessity of chemical crosslinking and enables multicomponent encapsulation of cargo with high encapsulation efficiency and content. With the combination of encapsulated drugs and an antigen incorporated in the shell, these novel nanocarriers show an enhancement in the immune response of cells.

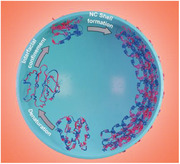

## 1 Introduction

Several challenges can prevent the translation of reported nanocarrier designs to clinical use. These hurdles include the (complex) chemistry required for their synthesis and manufacture, the use of toxic reagents, and the low encapsulation efficiency of therapeutic payloads.^[^ ^1^ ^]^ Hence, only a small number of nanocarrier designs have been approved by regulatory bodies such as the FDA (Food and Drug Administration) and EMA (European Medical Agency). Therefore, we designed a new class of drug‐loaded protein nanocapsules (NCs) without any chemical crosslinking but exploiting the intrinsic properties of proteins, namely denaturation leading to a shell formation without the use of toxic reagents and with a high encapsulation efficiency of the payloads.

The recent development of COVID‐19 mRNA vaccines using lipid nanoparticles has brought significant attention to the field of nanomedicine.^[^ ^2^ ^]^ One of the key reasons why these vaccines were able to be approved for use so quickly was the simplicity of their design. While lipid nanoparticles have proven to be the perfect fit for the delivery of mRNA, there are limitations to the delivery of other types of cargo using lipid nanoparticles, such as low encapsulation efficiency, uncontrolled leakage of cargo, etc. Even though polymeric nanoparticles can overcome some of these challenges, the synthesis process can be complex and time‐consuming.^[^ ^1^, ^3^ ^]^ However, naturally occurring polymers such as proteins are biocompatible, biodegradable, available in high abundance, low immunogenic, and non‐toxic.^[^ ^4^ ^]^ Therefore, we exploit the denaturation of proteins at droplet's interfaces by heating (“baking at the interface”) and the formation of disulfide bonds in proteins to form the protein NCs.^[^ ^5^ ^]^

We have previously reported different strategies for the synthesis of protein NCs containing water inside using the miniemulsion technique.^[^ ^6^ ^]^ This method enables the multicomponent encapsulation of cargoes with varying sizes and water solubility, with high encapsulation efficiency, making this technique superior to other nanocarrier synthesis approaches. So far, we used chemical cross‐linking of the proteins to form the protein shell.^[^ ^7^ ^]^ A novel and versatile strategy for the synthesis of protein NCs is presented here. Protein NCs were designed using different proteins such as hemoglobin (Hb) and keratin by interfacial denaturation (baking at the droplet interface) in an inverse miniemulsion. The baking process takes place at the oil–water interface where the protein unfolds to a more favorable conformation, which is also enhanced by heating.

This novel strategy utilizes abundantly available natural proteins, without any modifications to synthesize protein NCs. Moreover, the baking process at the droplet interface removes the necessity for additional chemical cross‐linking. Furthermore, we incorporated a model antigen ovalbumin in these protein NCs and encapsulated multiple cargoes; one imaging molecule, two adjuvants R848, and diABZI. These protein NCs showed excellent cell uptake and minimal cytotoxicity, and the successful delivery of adjuvants was proven by enhanced immune response. With the combination of encapsulated super‐additive adjuvants and an antigen (ovalbumin) incorporated into the protein shell, a novel nano vaccine for cancer immunotherapy was developed. The simplicity of the synthetic process along with the properties of the proteins would enable the speedy approval of these protein nanocarriers by regulatory bodies FDA and EMA.

## 2 Results and Discussion

**Figure 1:**
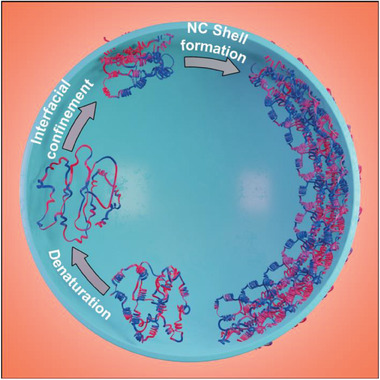
Illustration of protein nanocapsule (NC) formation in a water droplet after miniemulsification. Protein denatures upon heating or at the droplet interface and gets confined at the interface, where the majority of the hydrophobic amino acid residues in the proteins avoid water and get condensed at the interface leading to the formation of the protein shell. The blue color on the protein represents hydrophilic amino acid residues and red represents hydrophobic residues.

Protein NCs were synthesized using the inverse miniemulsion technique (Figure S1, Supporting Information).^[^ ^6a^ ^]^ Here, the inverse miniemulsion was formed by the aqueous protein solution as a dispersed phase and cyclohexane as a continuous phase. Poly((ethylene‐*co*‐butylene)‐*b*‐(ethylene oxide)) [P((E/B)‐b‐EO)] surfactant was used as surfactant and NaCl as the osmotic pressure agent. While forming the miniemulsion, the proteins start to get confined at the interface and the interfacial denaturation process is initiated to form stable protein NCs (**Figure** **1**). Previous reports suggest that the hydrophobic amino acid residues in the proteins avoid water, which drives the proteins towards a more condensed state at the oil–water interface, leading to interfacial denaturation.^[^ ^8^ ^]^ Moreover, as described by Suslick, ultrasonication creates superoxides from water and oxygen, which can also oxidize cysteines to form inter‐protein disulfide bridges.^[^ ^8^, ^9^ ^]^ To improve and enhance the formation of protein shells at the interface, the emulsion was heated at different temperatures (25, 40, or 60 °C) for 18 h. Proteins such as Hb denature upon heating, which is exploited in this step to form a rigid protein shell at the interface. After the formation of the protein NCs in cyclohexane, they were transferred to water.

**Figure 2:**
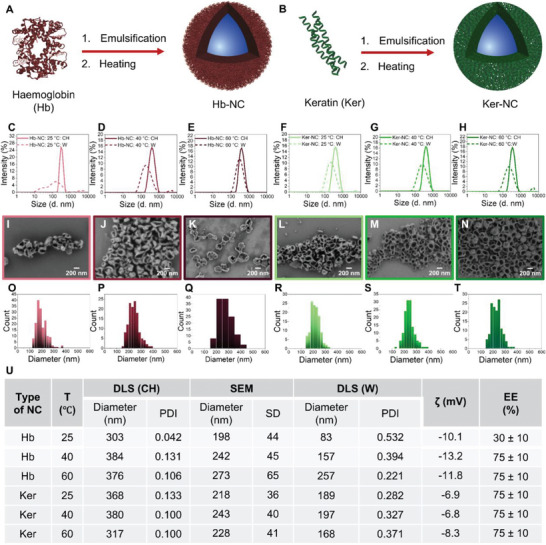
Synthesis of A) Hemoglobin nanocapsules (Hb‐NCs) and B) keratin nanocapsules (Ker‐NCs) from Hb and keratin by miniemulsification and heating. DLS measurements of C–E) Hb‐NC and F–H) Ker‐NC in cyclohexane (solid line) and water (dotted line) were obtained at different temperatures. SEM images of Hb‐NC were obtained at I) 25 °C, J) 40 °C, and K) 60 °C and Ker‐NCs were obtained at L) 25 °C, M) 40 °C and N) 60 °C showing a nanocapsule morphology. Size distributions obtained from SEM measurements for Hb‐NCs synthesized at O) 25 °C, P) 40 °C, and Q) 60 °C and Ker‐NCs synthesized at R) 25 °C, S) 40 °C and T) 60 °C. U) Table showing characterization of Hb‐NCs and Ker‐NCs synthesized at 25, 40 and 60 °C, T (temperature), CH (cyclohexane), W (water), SD (standard deviation), PDI (polydispersity index obtained from DLS measurements), ζ (zeta potential), mV(millivolts), EE (encapsulation efficiency of dye Cy5‐oligo (molecular weight ≈6.8 KDa)). Zeta potential was measured at pH 7.4.

The choice of the protein is important for the synthesis of protein NCs, as the proteins should be abundantly and easily available, cost‐effective, and with known properties. Hb is one such protein, which has 572 amino acids and a molecular weight of 65 KDa. Hb is abundantly available from bovine or human sources. Moreover, Hb has a low denaturation temperature of ≈60 °C. Therefore, the effect of temperature on the formation of Hb‐NCs was tested. Hb‐NCs were synthesized at 25, 40, and 60 °C. Hb‐NCs were characterized before and after transferring to water. DLS measurements showed narrow distributions of Hb‐NCs at all temperatures in cyclohexane with low polydispersity index (PDI, **Figure** **2**C–E,U). Moreover, scanning electron microscopy (SEM) images showed capsule morphology for all three Hb‐NCs (Figure 2I–K). SEM samples were prepared with Hb‐NCs in cyclohexane. Furthermore, a relatively larger protein shell was observed in SEM images of Hb‐NCs synthesized at 60 °C, this could be because of the higher denaturation of Hb at 60 °C. A narrow size distribution was also observed in SEM analysis, as can be seen from the histogram obtained (Figure 2O–Q). Transmission electron microscopy (TEM) analysis also showed capsule morphology (Figure S4, Supporting Information). The effect of temperature on the formation of Hb‐NCs was clearly observed after transferring them to water. As detected by DLS, a broad size distribution was obtained for Hb‐NCs synthesized at 25 °C (Figure 2C). The small size (83 nm) along with the large PDI (0.532) in water indicates the large disruption of Hb‐NCs. However, the broad distribution decreased with temperature, indicating the formation of a stable protein shell at higher temperatures (Figure 2D,E). The large size (257 nm) and relatively low PDI (0.221) showed the higher stability of Hb‐NCs synthesized at 60 °C. These results prove that the thermal denaturation provides higher stability of the Hb‐NCs formed.

For imaging purposes, Cy5‐oligo was encapsulated into these Hb‐NCs. Encapsulation of any cargo proceeds with the procedure explained above, where the cargo is dissolved in the dispersed phase along with the protein and the osmotic pressure agent (Figure S5, Supporting Information). If the cargo solubility is limited in water, cyclohexane‐immiscible solvents such as DMSO can be used to dissolve the cargo along with water. This also gives the possibility of encapsulating hydrophobic and hydrophilic cargoes in the same compartment. After sonication, the cargoes are retained in the emulsion droplets, because the cargoes are insoluble in the continuous phase cyclohexane. The formation of the protein shell upon temperature treatment limits the cargo from escaping the capsules even after transferring it to water. This provides very high encapsulation efficiency, which cannot be achieved by routinely used nanocarrier synthesis methods. The encapsulation efficiency was as high as 75 ± 10% for Hb‐NCs synthesized at 40 and 60 °C. However, the encapsulation efficiency was low (30 ± 10%) at 25 °C, which also shows the lower stability for the Hb‐NCs synthesized at lower temperatures. This higher encapsulation efficiency is the major advantage of this synthesis method.

To show the versatility of this synthesis strategy, as another abundantly available protein, keratin was chosen. Keratin is one of the least explored proteins for the purpose of nanocarrier design. It is a structural protein found in mammalian tissues such as hair, wool, skin, nails, and horns and in birds (bird beak and feathers), hence it is highly abundant and cheap.^[^ ^10^ ^]^ The high strength of hair, wool, etc. is predominantly because of the numerous disulfide bonds present in keratin. However, because of the same reason, keratins are insoluble in water. Disulfide bonds are partially cleaved and sulfonated to make them water‐soluble. The denaturation temperature of keratin is >100 °C,^[^ ^10b^ ^]^ hence thermal denaturation cannot be achieved in this synthesis method. Nevertheless, keratin NCs (Ker‐NCs) synthesis was performed at higher temperatures as well. As expected, no significant differences were observed in the properties of Ker‐NCs synthesized at 25, 40, and 60 °C (Figure 2F–H). Therefore, the nanocapsule formation relies completely on the denaturation process at the water‐cyclohexane interface and/or the formation of disulfide bonds. DLS in cyclohexane showed the narrow distribution of Ker‐NCs at all temperatures, with low PDI (≈0.1). SEM and TEM images proved the temperature‐independent formation of NCs (Figure 2L–N; Figure S7, Supporting Information). A narrow distribution of the NCs can be also seen from the histograms obtained from SEM analysis (Figure 2R–T). As opposed to the case of Hb‐NCs, no significant difference was observed among all three Ker‐NCs after transferring them to the water phase (Figure 2F–H) confirming that there is no temperature‐dependence for the formation of Ker‐NCs due to higher denaturation temperature of keratin. The higher stability of Ker‐NCs even without thermal denaturation can be attributed to the presence of a large fraction of disulfides present in them, which can be broken, and inter‐keratin disulfide bonds can be formed upon ultrasonication. A high encapsulation efficiency of 75 ± 10% for Cy5‐oligo at all the temperatures used also proves the non‐dependence on temperature for the synthesis of Ker‐NCs. As there is no requirement for heating for the synthesis of Ker‐NCs, it can be used for the encapsulation and delivery of heat‐sensitive cargoes.

**Figure 3:**
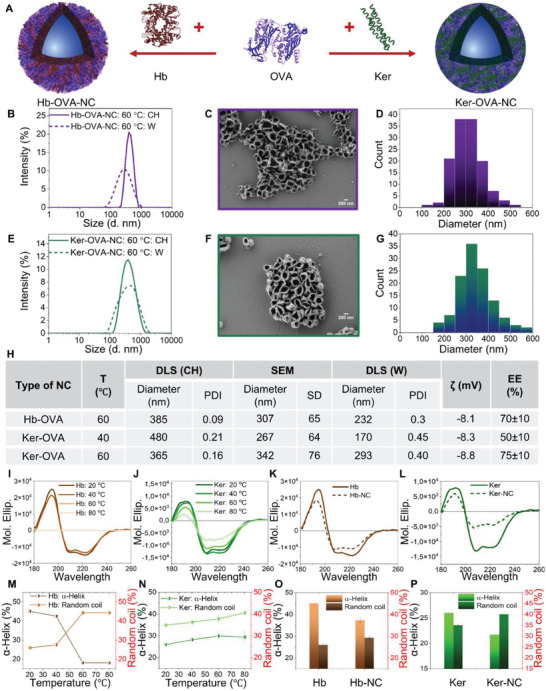
A) Synthesis of hemoglobin‐ovalbumin nanocapsules (Hb‐OVA‐NCs) and keratin‐ovalbumin nanocapsules (Ker‐OVA‐NCs). Hb and OVA were dissolved in the dispersed phase (water) in a 1:1 ratio and emulsified with surfactant in cyclohexane, emulsion was heated to obtain Hb‐OVA‐NCs. Similarly, in the case of Ker‐OVA‐NCs, keratin, and OVA were dissolved in water in a 1:1 ratio. DLS measurements of B) Hb‐OVA‐NCs and E) Ker‐OVA‐NCs in cyclohexane and water (synthesis temperature: 60 °C). SEM image C) Hb‐OVA‐NCs and F) Ker‐OVA‐NCs obtained at 60 °C, D–G) corresponding size distribution from SEM measurements. H) Table showing characterization of Hb‐OVA‐NCs and Ker‐OVA‐NCs synthesized at 40 and 60 °C. T (temperature), CH (cyclohexane), W (water), SD (standard deviation), PDI (polydispersity index obtained from DLS measurements), ζ (zeta potential), mV (millivolts), EE (encapsulation efficiency of dye Cy5‐oligo (molecular weight ≈6.8 KDa)). Zeta potential was measured at pH 7.4. Circular Dichroism (CD) spectra of I) Hb, J) keratin measured at different temperatures. CD spectra of K) Hb and Hb‐NCs, L) Keratin and Ker‐NCs measured at 20 °C. Hb‐NCs and Ker‐NCs were synthesized at 60 °C. Percentage of α‐helix and random coil obtained from CD measurements for M) Hb and N) keratin at different temperatures. Percentage of α‐helix and random coil obtained from CD measurements for O) Hb‐ and Hb‐NCs, P) Keratin and Ker‐NCs measured at 20 °C.

Circular dichroism (CD) spectra of Hb and keratin were recorded at different temperatures to see the influence on the secondary structures with temperatures in the interfacial denaturation process. In general, upon denaturation, the percentage of α‐helix decreases, and the percentage of random coil increases in a protein.^[^ ^11^ ^]^ In the case of Hb, only a slight change in the α‐helix % and random coil % was observed from 20 to 40 °C (**Figure** **3**I,M). At 60 and 80 °C, Hb was completely denatured as detected by the decrease in the α‐helix % (Figure 3M). The signal for Hb at 60 and 80 °C was barely detectable (Figure 3I; Figure S8A, Supporting Information). For the keratin, no significant changes in CD spectra were observed with temperature up to 80 °C, and the percentage of α‐helix remained constant with temperature (Figure 3J,N). This proves that thermal denaturation is not observed within this temperature range in the case of keratin. In order to check what happens after the formation of Hb‐NCs and Ker‐NCs, CD spectra were recorded at 20 °C for Hb, keratin, Hb‐NCs, and Ker‐NCs synthesized at 60 °C. Even though the synthesis of Hb‐NCs was carried out at 60 °C, there was only partial denaturation of Hb observed (Figure 3K,O). Compared to pure Hb, the decrease in α‐helix was only ≈10% which is due to the confinement of Hb at the oil–water interface, which limits further thermal denaturation of Hb. There was no thermal denaturation observed for pure keratin, however, CD analysis of Ker‐NCs revealed a decrease in α‐helix % and an increase in random coil % (Figure 3L,P) which is due to the interfacial denaturation of keratin while forming Ker‐NCs. Interfacial denaturation was previously shown by Avivi and Gedanken for the case of microspheres formed using streptavidin. The authors proposed that the hydrophobic amino acid residues avoid water which drives the proteins toward a more condensed state at the oil–water interface.^[^ ^8^ ^]^ Another commonly used method for the analysis of protein denaturation is nano‐differential scanning fluorimeter (nano DSF).^[^ ^12^ ^]^ There was no change observed for keratin and Ker‐NCs, due to the lack of thermal denaturation (Figure S9, Supporting Information). For Hb, a change was observed at ≈65 °C, which is close to the denaturation temperature of Hb (Figure S10, Supporting Information). For Hb‐NCs, no change was observed which might be because Hb in Hb‐NCs are already partially denatured while forming Hb‐NCs.

For the effective development of anti‐tumor nano vaccines via dendritic cell (DC) mediated T cell activation, there are multiple steps that need to be taken care of: antigen and adjuvant uptake by DCs, antigen presentation by DCs, release of cytokines and expression of costimulatory marker molecules.^[^ ^13^ ^]^ DCs are the most potent antigen‐presenting cell population which are extensively targeted for vaccine development. The first step is to incorporate the model antigen OVA into protein NCs for the enhanced uptake of the antigen by DCs. OVA has been widely used as a model antigen as it was the first protein for which the loading of peptides onto MHC‐I and MHC‐II was demonstrated. Also, the stimulation of T cells was investigated with OVA for the first time. As a non‐mammalian antigen, it also elicits clearly defined immune reactions and there are even mouse strains where T cells are genetically engineered such that they specifically recognize these peptide‐MHC complexes. OVA as an antigen is therefore a benchmark protein in many vaccination studies. The first effort was to synthesize OVA‐NCs using the interfacial denaturation of OVA in an inverse miniemulsion, as previously described in the case of Hb‐NCs (Figure 1). Even though OVA‐NCs were formed at 40 and 60 °C as seen in the SEM images (Figure S11, Supporting Information), their long‐term stability and reproducibility were limited. To overcome this, OVA was incorporated into Hb‐NCs and Ker‐NCs by using a mixture of proteins to give Hb‐OVA‐NCs and Ker‐OVA‐NCs respectively (Figure 3A). Since, similar to Hb, OVA can be also denatured upon heating, Hb‐OVA‐NCs were synthesized at 60 °C. DLS showed a size of 232 nm after transferring to water and SEM and TEM images showing capsule morphology corroborated the formation of NCs (Figure 3B,C and Figure S14, Supporting Information). Moreover, Hb‐OVA‐NCs also showed a high encapsulation efficiency of 70 ± 10% for Cy5‐oligo. As the Ker‐NCs formed independent of the temperature, the Ker‐OVA‐NC synthesis was performed at two different temperatures; 40 and 60 °C. While at 60 °C, Ker‐OVA‐NC remained stable even after transferring to water (Figure 3E), at 40 °C, the stability was low, as seen from the bimodal distribution observed in DLS measurements after transferring to water (Figure S15, Supporting Information). The same result was corroborated by the slightly lower encapsulation efficiency (50 ± 10%) obtained for Ker‐OVA‐NCs synthesized at 40 °C, whereas a high encapsulation efficiency of 75 ± 10% was obtained for Ker‐OVA‐NCs synthesized at 60 °C.

**Figure 4:**
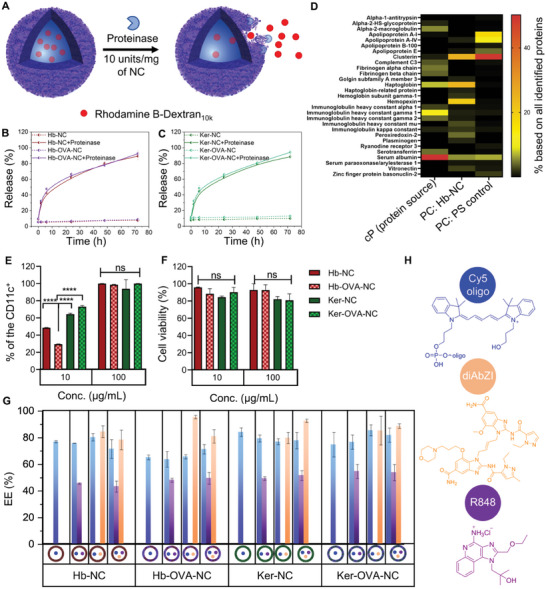
Schematic illustration of degradation and release of cargoes from protein NC in the presence of proteinase K. Protein NC containing RhodamineB‐Dextran_10K_ in PBS (pH 7.4) buffer was incubated with 10 units mg^−1^ of enzyme. Aliquots at regular intervals were taken and the released cargo was retrieved using centrifugal filtration. Fluorescence was measured to calculate the percentage of released cargo. Release profile of B) Hb‐NC, Hb‐OVA‐NC, C) Ker‐NC, Ker‐OVA‐NC. D) Proteomic analysis of the protein corona (PC) composition. The heat map shows the relative amount (> 1%) of the most abundant proteins. PC was desorbed from Hb‐NCs and control polystyrene NPs (PS control). NCs and NPs were incubated in citrate human citrate plasma (cP) for 1 h at 37 °C, subsequently centrifuged and washed 3 times with PBS and the remaining adsorbed proteins were desorbed with 2% w/v SDS. Protein composition for cP as an initial protein source is presented for comparison. Protein NC E) uptake experiment and F) cytotoxicity experiment performed on bone marrow‐derived dendritic cells (BMDCs) with two different concentrations (10 and 100 µg mL^−1^) for 24 h (mean ± SD; *n* = 3). Statistical analyses were performed using one‐way ANOVA with Tukey's post hoc test. (ns, not statistically significant; ^****^ *p* ≤ 0.0001). G) Encapsulation efficiency (EE) of different cargoes Cy5‐oligo (blue), diAbZI (orange), and R848 (purple) in Hb‐, Ker‐, Hb‐OVA, and Ker‐OVA‐NCs. H) Chemical structures of Cy5‐oligo, diAbZI, and R848 (oligo sequence: CCACUCCUUUCCAGAAAACU).

Another inherent advantage of protein NCs is their biodegradation in the presence of enzymes and thereby release of the cargo. For this purpose, the model cargo rhodamine B‐Dextran_10k_ was encapsulated in Hb‐, Ker‐, Hb‐OVA‐ and Ker‐OVA‐NCs and its release in the presence of a protease enzyme proteinase K was evaluated (**Figure** **4**A). Release studies of the cargo in the presence and absence of 10 units enzymes mg^−1^ of NC showed an enhanced release of cargo from all four protein NCs (Figure 4B,C). In the absence of the enzyme, only ˂10% of the cargo was released even after 72 h. On the other hand, in the presence of the enzyme, ≈50% of the cargo was released within 6 h and then a steady release was observed for 72 h to achieve ≈90% release.

To further characterize the protein NCs in biologically relevant media, we incubated them in human citrate plasma (cP) and subjected them to a protein corona (PC) preparation workflow with subsequent quantitative analysis by Pierce 660 nm protein assay and an LC‐MS analysis to reveal the PC composition. We used a gentle centrifugation‐based separation process to remove non‐bound or loosely‐bound proteins to isolate the hard PC and preserve NC stability. As a control, well‐studied polystyrene nanoparticles (PS‐NP) were used.^[^ ^14^ ^]^ As seen in Figure S22B (ISupporting Information), the amount of desorbed corona proteins was the highest for Hb‐NCs, however, LC‐MS analysis revealed a high fraction (86.2%) of Hb derived from Hb‐NCs themselves because of the NC disruption in the desorption step (2% SDS (w/v) buffer and 5 min 95 °C). Hence the PC on Hb‐NCs was obtained after subtracting the fraction of capsule‐derived Hb, which was higher than the protein background from human plasma (mock PC). However, for Ker‐NCs the PC preparation process was either too harsh (hence resulting capsule debris does not sediment and is lost during washing steps) or too little centrifugation force was used to enable sedimentation of the Ker‐NCs, since the desorbed protein amount was in the range of the control. This was also evident after subjecting the samples to SDS‐PAGE and a silver staining (Figure S22A, Supporting Information). The gel lanes for the PC from Hb‐NCs illustrated a more diverse protein pattern with additional bands different from the initial Hb starting material (Hb control) indicating the corona proteins. Especially, a band slightly above 38 kDa accounts for the enrichment of clusterin, a chaperone known to be the main PC constituent of PS‐NPs.^[^ ^14^ ^]^ Due to the low protein amount for the PC from Ker‐NCs, we only subjected the PC samples for Hb‐NCs, PS‐NPs, and the cP as protein sources for LC‐MS analysis to reveal the protein composition. As evident in the heatmap for the most abundant proteins (Figure 4D), the PC profile for PS‐NPs appears with a characteristic and high amount of lipoproteins, with apolipoprotein A‐I and clusterin being the most abundant ones, as described previously by our group.^[^ ^14a^ ^]^ For Hb‐NCs, besides clusterin (25.3%), also haptoglobin (24.4%) and hemopexin (20.1%) were enriched in the hard protein corona. The latter two are already well known to interact with Hb and its heme group, also under physiological conditions.^[^ ^15^ ^]^

In vitro uptake studies demonstrated that protein NCs exhibited excellent uptake in bone marrow‐derived dendritic cells (BMDCs) (Figure 4E), while inducing only minute levels of cytotoxicity (Figure 4F). These findings suggest that protein NCs have the potential to be utilized as an effective delivery system for antigens to BMDCs, which are crucial for the initiation of immune responses. Moreover, the low cytotoxicity of NCs toward BMDCs indicates their safety for use in therapeutic applications.

Cancer is a complex and multifactorial disease that affects millions of people worldwide. Immunotherapy has emerged as a promising approach to cancer treatment, as it harnesses the patient's own immune system to fight cancer cells. One approach to cancer immunotherapy is the use of cancer vaccines, using tumor cell‐associated antigens, to awaken the body's immune system against tumor cells. Several studies have already shown that Toll‐like receptor 7 and 8 (TLR7/8) agonist R848 or STING agonist diABZI‐loaded nanovaccines can elicit strong immune responses to achieve the desired effect of cancer treatment.^[^ ^16^ ^]^ Moreover, the combination of STING and TLR 7/8 agonists could enhance the immune response to cancer vaccines, leading to better outcomes for cancer patients.^[^ ^17^ ^]^ Hence, these two adjuvants (R848 and diABZI) were encapsulated along with the imaging molecule Cy5‐oligo. Due to the low solubility of R848 and diABZI in water, they were dissolved in DMSO and added to the dispersed phase which already contains the proteins, NaCl and Cy5‐oligo in water. The possibility of using DMSO allows for an increase in the loading capacity of these cargoes. Since DMSO and cyclohexane are immiscible, the adjuvants are trapped in the aqueous droplet while forming the nanocapsules, which ensures high encapsulation efficiency. Moreover, these adjuvants are insoluble in cyclohexane (oil phase), hence they do not partition into the oil phase during emulsion formation and purification. The encapsulation efficiency of the adjuvants was measured using their inherent fluorescence. A high encapsulation efficiency of 70–80% was observed for Cy5‐oligo in all four NC systems (Figure 4G). While >80% encapsulation efficiency was obtained for diABZI, a slightly lower encapsulation efficiency of ≈50% was observed for R848. The slightly lower encapsulation efficiency of R848 might be due to the smaller molecular size. Nevertheless, a high retention of all the cargo was achieved.

**Figure 5:**
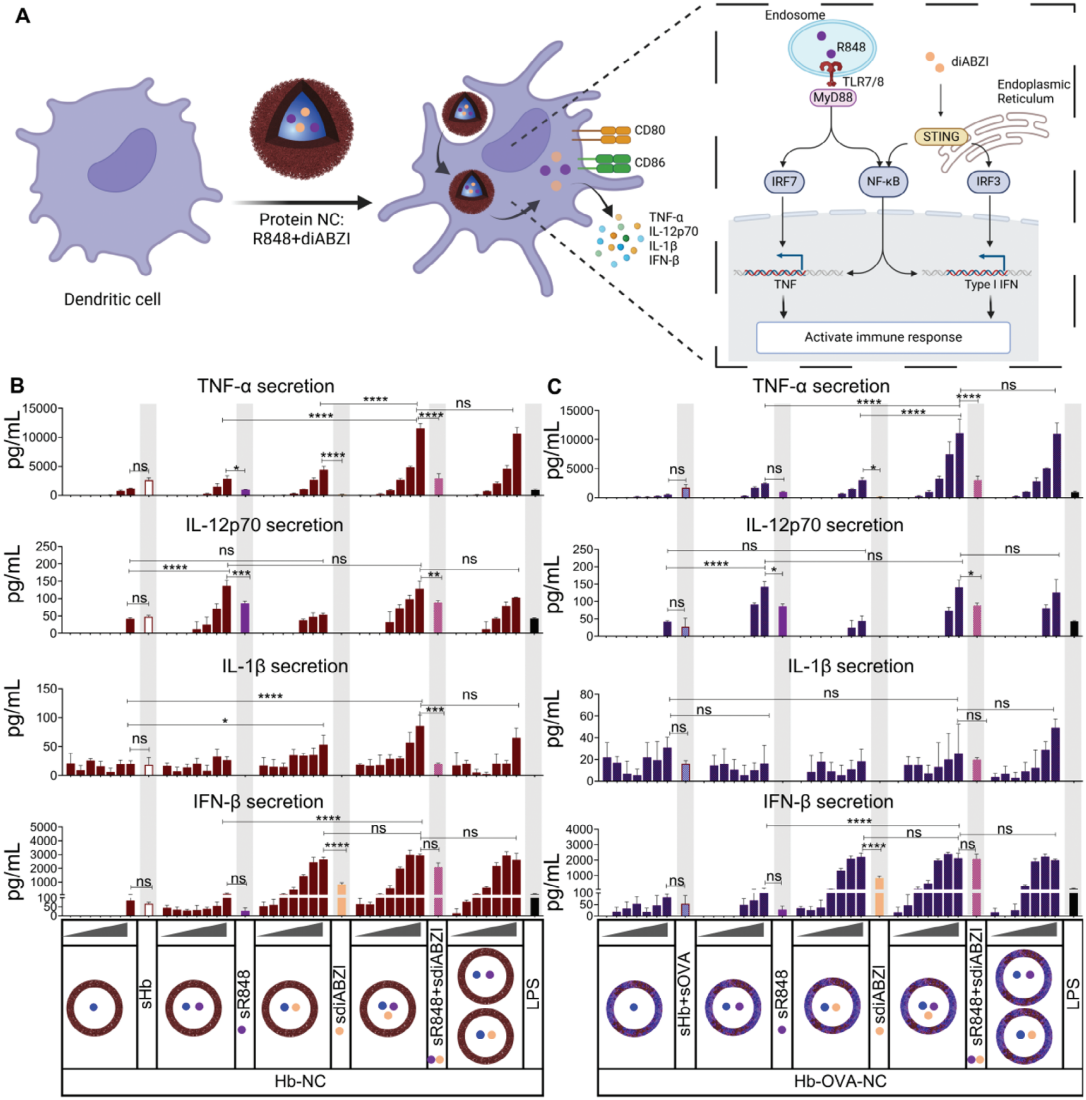
Evaluation of the efficacy of adjuvants loaded Hb‐NCs and Hb‐OVA‐NCs in inducing cytokine secretion by bone marrow‐derived dendritic cells (BMDCs). To achieve this, BMDCs were exposed to varying concentrations (0.1, 0.3, 1, 3, 10, 30, 100 µg mL^−1^) of empty or adjuvant‐loaded Hb‐NCs and Hb‐OVA‐NCs, soluble hemoglobin (sHb), soluble ovalbumin (sOVA), sHb + sOVA, soluble R848 (sR848, 600 ng mL^−1^), soluble diABZI (sdiABZI, 1.1 µg mL^−1^), or LPS (100 ng mL^−1^) for 24 h. The concentration of soluble adjuvants used for treatment was equivalent to the concentration present in 100 µg mL^−1^ of Hb‐NCs. A) Schematic illustration (created using BioRender) of how adjuvants loaded protein NC improve the function of BMDCs. The supernatants of BMDCs were collected to detect TNF‐α and IL‐12p70, IL‐1β, and IFN‐β expression (mean ± SD; *n* = 3). B) Hb‐NCs and C) Hb‐OVA‐NCs. All statistical analyses were performed using one‐way ANOVA with Tukey's post hoc test. (ns, not statistically significant ^*^ *p* ≤ 0.05; ^**^ *p* ≤ 0.01; ^***^ *p* ≤ 0.001; ^****^ *p* ≤ 0.0001).

**Figure 6:**
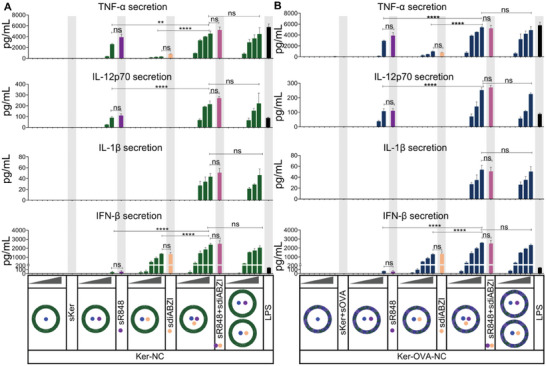
Evaluation of the efficacy of adjuvants‐loaded Ker‐NCs and Ker‐OVA‐NCs in inducing cytokine secretion by BMDCs. To achieve this, BMDCs were exposed to varying concentrations (0.1, 0.3, 1, 3, 10, 30, 100 µg mL^−1^) of empty or adjuvant‐loaded Ker‐NCs and Ker‐OVA‐NCs, soluble keratin (scar), soluble ovalbumin (sOVA), sKer+sOVA, soluble R848 (sR848, 600 ng mL^−1^), soluble diABZI (sdiABZI, 1.1 µg mL^−1^), or LPS (100 ng mL^−1^) for 24 h. The concentration of soluble adjuvants used for treatment was equivalent to the amount present in 100 µg mL^−1^ of Ker‐NCs. The supernatants of BMDCs were collected to analyze TNF‐α, IL‐12, IL‐1β, and IFN‐β secretion (mean ± SD; *n* = 3). A) Ker‐NCs and B) Ker‐OVA‐NCs. All statistical analyses were performed using one‐way ANOVA with Tukey's post hoc test. (ns, not statistically significant; ^**^ *p* ≤ 0.01; ^****^ *p* ≤ 0.0001).

To assess the ability of adjuvant‐loaded protein NC to stimulate BMDCs, cytokine secretion and expression of CD80 and CD86 co‐stimulatory molecules were measured after 24 h of incubation with various protein NC formulations and compared to the respective soluble adjuvants. Lipopolysaccharide (LPS), a potent TLR4 agonist known to induce high expression of DC maturation, was used as a positive control.^[^ ^18^ ^]^ DCs, macrophages, and B cells act as antigen‐presenting cells (APCs) presenting antigens to T cells, thus facilitating their recognition by the immune system. DCs are highly effective APCs so efforts to generate therapeutic immunity against cancer focus on targeting DCs.^[^ ^19^ ^]^ Inflammatory cytokines secreted by DCs, including TNF‐α, IL‐1β, IL‐12p70, and type 1 interferon (IFN‐α/β), which play a crucial role in promoting the proliferation of CD4+ T cells and CD8+ T cells, are closely linked to vaccine efficacy.^[^ ^20^ ^]^ R848 is a potent synthetic agonist of TLR7/8 which activates the immune system through the production of cytokines such as TNF‐α and interleukin 12 (IL‐12).^[^ ^21^ ^]^ In addition, stimulation of immune cells with the STING agonist diABZI has been shown to induce the production of cytokines, which are key mediators of immune responses.^[^ ^22^ ^]^ Given that (Figure 4F) protein NC (Hb‐NCs, Hb‐OVA‐NCs, Ker‐NC, and Ker‐OVA‐NCs) were taken up by the BMDCs, we then evaluated cytokine secretion by the BMDCs. The aim was to determine if there was a synergistic effect when the adjuvants were used in combination and if the adjuvants could be released from the protein NCs and subsequently secret cytokines in BMDCs. The results showed that both Hb‐NCs and Hb‐OVA‐NCs without payload elicited a notable upregulation in the expression of cytokines, a phenomenon also observed in soluble hemoglobin (sHb) and soluble Hb combined soluble ovalbumin (sHb+sOVA) (**Figure** **5**B,C). Nevertheless, such a response was not observed in soluble keratin (sKer) and soluble keratin in combination with soluble ovalbumin (sKer+sOVA) (**Figure** **6**), suggesting that Hb possesses inherent immunostimulatory capabilities. Consistent with our expectations, treatment of BMDCs with soluble R848 led to a higher expression level of TNF‐α and IL‐12p70 in comparison to soluble diABZI treatment, whereas treatment with soluble diABZI resulted in a greater upregulation of IFN‐β relative to sR848 (Figures 5B,C and 6). Additionally, we observed that the expression of TNF‐α was significantly higher in all protein NCs loaded with both R484 and diABZI as compared to protein NCs loaded with single adjuvants (Figure 5B,C and Figure 6). This indicates that the combination of R848 and diABZI can synergistically enhance TNF‐α expression, which are pivotal cytokines known to regulate immune responses,^[^ ^23^ ^]^ this highlights the potential therapeutic benefits of the combination. Significant or moderate increases in the expression levels of TNF‐α and IL‐12p70 were also observed in Hb‐NCs and Hb‐OVA‐NCs loaded with single or combined adjuvants compared to their corresponding soluble adjuvants (Figure 5B,C). Moreover, a concentration‐dependent increase in TNF‐α and IL‐12p70 secretion was observed in both Hb‐NCs and Hb‐OVA‐NCs. IL‐12 serves as a crucial regulator of the Th1‐mediated immune response against cancer,^[^ ^24^ ^]^ by stimulating the proliferation and cytotoxic activity of activated NK cells and CD8^+^ T cells, as well as enhancing the secretion of IFN‐γ by these cells. Additionally, IL‐12 promotes the differentiation of CD4^+^ T cells toward the Th1 phenotype, which is essential for effective anti‐tumor immunity.^[^ ^17^ ^]^ We did not detect IL‐1β expression in response to LPS, sR848, and sdiABZI alone. However, we observed a detectable level of IL‐1β expression upon combined stimulation with R848 and diABZI adjuvants, indicating that the effects of the adjuvants were synergistic (Figures 5B,C and 6). Altogether, the comparative analysis of immunostimulatory capacity between Ker‐NC and Ker‐OVA‐NC, and Hb‐NC and Hb‐OVA‐NC demonstrated that the latter two can augment the potency of adjuvants. Therefore, the use of Hb‐NCs and Hb‐OVA‐NCs as carriers for adjuvants may lead to a more robust and effective immune response. Some studies have demonstrated that Hb and LPS exhibit a synergistic effect on the production of inflammatory cytokines in immune cells, potentially due to Hb's ability to modulate innate immune response.

**Figure 7:**
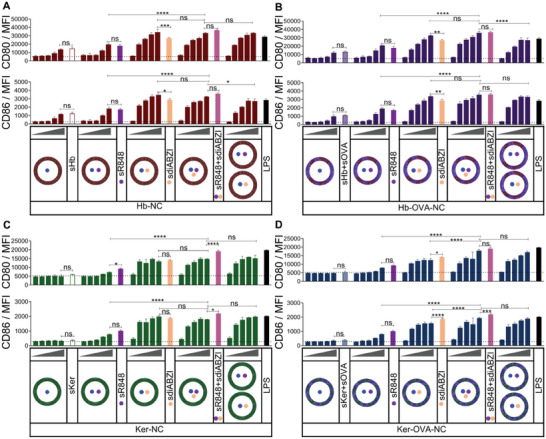
Evaluation of DC maturation markers CD80 and CD86 following stimulation with adjuvant‐loaded protein NC. To achieve this, BMDCs were exposed to varying concentrations (0.1, 0.3, 1, 3, 10, 30 µg mL^−1^) of empty or adjuvant‐loaded protein NC, soluble proteins, soluble R848 (sR848, 200 ng mL^−1^), soluble diABZI (sdiABZI, 333 ng mL^−1^), or LPS (100 ng mL^−1^) for 24 h. The concentration of soluble adjuvants used for treatment was equivalent to the amount present in 30 µg mL^−1^ of protein NC. The surface expression of CD80 and CD86 in BMDCs treated with various adjuvants‐loaded protein NC was determined by flow cytometry based on median fluorescence intensity (MFI). A) Hb‐NC, B) Hb‐OVA‐NC, C) Ker‐NC D) Ker‐OVA‐NC. Dashed lines indicate expression levels of the untreated control (mean ± SD; *n* = 3). All statistical analyses were performed using one‐way ANOVA with Tukey's post hoc test. (ns, not statically significant ^*^ *p* ≤ 0.05; ^**^ *p* ≤ 0.01; ^****^ *p* ≤ 0.0001).

Next, the expression of costimulatory surface molecules in BMDCs was assessed. Consistent with the cytokine expression results, the combined application of soluble R848 and soluble diABZI resulted in a significant upregulation of surface markers compared to the use of soluble R848 alone. Moreover, the combined use of R848 and diABZI demonstrated a significant or moderate upregulation when compared to the use of single adjuvants (**Figure** **7**). In contrast to BMDCs treated with empty Ker‐NCs or Ker‐OVA‐NCs, these results demonstrated a higher expression of surface markers using empty Hb‐NCs, Hb‐OVA‐NCs, or their corresponding soluble forms, consistent with the cytokine expression results. These findings also reveal that the sole administration of diABZI‐loaded Hb‐NCs and Hb‐OVA‐NCs induced a higher upregulation of CD80 and CD86 expression, compared to treatment with the soluble diABZI (Figure 7A,B). Furthermore, we observed that only the highest concentration of R848‐loaded protein NCs administered to BMDCs resulted in a significant upregulation of both markers, in comparison to their corresponding empty NCs. Consistent with the results obtained from cytokine expression analysis (Figure 6A,B), the application of single or combined adjuvant‐loaded Ker/Ker‐OVA‐NCs did not exhibit a significant difference in terms of BMDC maturation, when compared to the use of soluble adjuvants (Figure 7C,D). To evaluate the stability of the protein NCs, a biological activity assay was conducted on both the protein NCs and their corresponding supernatants (after centrifuge filtration using an Amicon filter) four weeks after synthesis, with equivalent volumes. The levels of cytokine secretion were then compared to determine any differences in stability (Figure S23, Supporting Information). Given that, R848 and diABZI induce TNF‐α and IFN‐β production, respectively, here we only showed the expressions of these two cytokines. We observed only a slight TNF‐α secretion solely in the case of supernatants derived from Hb/Hb‐OVA formulations, which is even weaker than that induced by corresponding empty Hb/Hb‐OVA‐NCs. For the case of Ker and Ker‐OVA‐NCs, the supernatant induced no secretion of TNF‐α. Moreover, no IFN‐β secretion was observed in any case. This showed the excellent stability of the protein NC even after storage for four weeks.

## 3 Conclusion

In summary, utilizing the inverse miniemulsion technique, a novel class of protein nanocapsules was synthesized from hemoglobin and keratin. The nanocapsule shell was formed by denaturation and disulfide bond formation, without additional reagents such as crosslinkers or other chemical modifications. While the hemoglobin nanocapsule synthesis was temperature‐dependent the keratin nanocapsule synthesis was independent of temperature. As a model antigen ovalbumin was incorporated in a protein shell and two adjuvants for cancer immunotherapy were encapsulated with high encapsulation efficiency. The nanocapsules could be enzymatically cleaved to allow cargo release. In vitro studies of the dual adjuvant encapsulated protein NC showed their capacity to enhance the immune response. This novel strategy opens up new a way of formulating drug‐loaded protein nanocapsules without any chemical crosslinking, but by denaturation, without the use of toxic reagents, and with a high encapsulation efficiency of therapeutic cargoes.

## 4 Experimental Section

### Synthesis of Protein Nanocapsules (NC)

The synthesis of protein NC is described taking hemoglobin nanocapsules (Hb‐NCs) as an example. The continuous phase was prepared by dissolving 35.7 mg P((E/B)‐b‐EO) in 7.5 g cyclohexane by sonication at 40 °C in a 40 mL vial and the dispersed phase was prepared by dissolving 50 mg Hb, 7.2 mg NaCl and 50 µL Cy5‐oligo (136 µM) in 450 µL sterile water using thermoshaker (500 rpm, 20 °C). The continuous phase was added to the dispersed phase and stirred at 750 rpm for 10 min and then ultra‐sonicated for 180 s (20 s ultrasonication, 10 s pause) with water cooling at 70% amplitude using a Branson 450 W sonifier and a 1/2′ tip. After the sonication, 10.7 mg P((E/B)‐b‐EO) in 5 g cyclohexane was added to the miniemulsion dropwise at 25 °C and then stirred at 25 or 40 or 60 °C for 18 h. Hb‐NCs in cyclohexane was purified using centrifugation. 8 mL of the NC dispersion was taken in two 15 mL centrifuge tubes and centrifuged at 1500 g for 30 min at 20 °C. After centrifugation, the supernatant (supernatant 1) was transferred to another set of 15 mL centrifuge tubes, and the pellet was redispersed after adding 8 mL cyclohexane by shaking the tubes for 1 min in a sonication bath. The centrifugation step was repeated at 1500 g for 30 min at 20 °C. To increase the yield, supernatant 1 was centrifuged at 3000 g for 30 min at 20 °C. After centrifugation, the supernatant was disposed and the centrifugation was repeated after redispersing in 8 mL cyclohexane. Hb‐NCs from all the centrifuge tubes were redispersed in cyclohexane by pipetting to make a final volume of 1 mL. To transfer Hb‐NCs from cyclohexane to water, 500 µL of purified NC dispersion in cyclohexane was added dropwise with stirring to 5 mL SDS solution (0.1 wt.%) in a 40 mL glass vial at 750 rpm and then vigorously shaken in circle motions in an ultrasound bath (25 kHz) for 5 min. Afterwards, the vial was completely covered with aluminum foil, and a few holes were punched into the foil with a needle at the glass opening and the dispersion was stirred for 18 h at room temperature at 750 rpm to evaporate cyclohexane. Before purification in water, the dispersion in water was shaken in the ultrasonication bath in circle motions for 1 min, then transferred to a 40 mL Amicon centrifuge filter 50 KDa and centrifuged at 800 g for 30 min at 20 °C. The supernatant was removed and the dispersion was redispersed in 5 mL water. The centrifugation step was repeated three times and the purified capsule dispersion (≈2 mL) was transferred to a 4 mL glass vial and stored at 4 °C.

Other types of protein NCs were prepared following the same protocol, the only difference is in the preparation of the dispersed phase. The dispersed phase composition of each protein NC sample is the following. 1) Ovalbumin nanocapsules (OVA NCs): 50 mg OVA, 7.2 mg NaCl, 50 µL Cy5‐oligo (136 µm) and 450 µL water. 2) Keratin nanocapsules (Ker‐NC): 500 µL (≈25 mg) keratin (partially sulfonated, 5% in Water), 7.2 mg NaCl and 50 µL Cy5‐oligo (136 µm). 3) Hemoglobin‐ovalbumin nanocapsules (Hb‐OVA‐NCs): 25 mg Hb, 25 mg OVA, 7.2 mg NaCl, 50 µL Cy5‐oligo (136 µm) and 450 µL water. 4) Keratin‐ovalbumin nanocapsules (Ker‐OVA‐NCs): 400 µL (≈20 mg) keratin (partially sulfonated, 5% in Water), 20 mg OVA, 7.2 mg NaCl, 50 µL Cy5‐oligo (136 µm), 50 µL water.

### Characterization of the Protein‐Nanocapsules

Dynamic light scattering (DLS) was used to determine the average size and polydispersity (PDI) of the NCs. The NCs were diluted (2 µL sample in 300 µL in cyclohexane or 50 µL NC in water diluted in 300 µL H_2_O) was measured on a Malvern Zetasizer Nano S (Malvern Panalytical) equipped with a detector at 90° scattering mode at 20 °C for cyclohexane and 25 °C for water. The zeta potential of the NCs was measured in PBS buffer pH 7.4 with a Zetasizer (Malvern Panalytical) at 25 °C. Scanning electron microscopy (SEM) images were captured using a field emission microscope (LEO (Zeiss) 1530 Gemini, Oberkochen, Germany) working at an accelerating voltage of 170 V. 2 µL of the diluted NC solution in cyclohexane (dilution same as samples for DLS) was dropped onto the silica wafers and dried at room temperature. Transmission electron microscopy (TEM) images were captured using a Jeol 1400 transmission electron microscope with a voltage of 120 kV. Samples were prepared by placing a drop of the NC dispersion onto a 300 mesh carbon‐coated copper grid and drying at room temperature. Fluorescence was measured using a microplate reader (Infinite M1000, Tecan, Switzerland). Circular dichroism (CD) spectroscopy of the proteins was measured using a JASCO 1500 CD instrument. Samples of 0.1 mg mL^−1^ concentration were taken in a 0.1 cm quartz cuvette and measured from 260 to 180 nm. JWMVS‐529 Multivariate SSE (Secondary Structure Estimation) analysis program included in JASCO was used to analyze the secondary structures of the proteins. Proteins and protein NCs were also characterized using nanoDSF, they were loaded into nanoDSF high sensitivity capillaries (NanoTemper Technologies) and applied into a Prometheus NT·48 instrument. A linear thermal ramp program starting 20 to 90 °C (1 °C min^−1^) was set and the tryptophan fluorescence was measured at 330 and 350 nm.

### Determination of the Encapsulation Efficiency

NCs after transferring to 0.1 wt.% SDS were concentrated using Amicon centrifuge filter 50 KDa for 30 min at 800 g at 20 °C and then washed three times with water by centrifuging for 30 min at 800 g at 20 °C. The encapsulation efficiency was determined by measuring the fluorescence using a microplate reader (Infinite M1000 Tecan, Switzerland) of the non‐purified and purified NCs.

### Encapsulation and Release of Rhodamine B Isothiocyanate–Dextran_10k_

Rhodamine B isothiocyanate–Dextran_10k_ was encapsulated in Hb, Hb‐OVA, Ker, and Ker‐OVA‐NCs following the procedures shown above, where rhodamine B isothiocyanate–Dextran_10k_ was dissolved in the dispersed phase. NCs after transferring to 0.1 wt.% SDS was concentrated using Amicon centrifuge filter 50 KDa for 30 min at 800 g at 20 °C and then washed three times with water by centrifuging for 30 min at 800 g at 20 °C. The solid content was measured by evaporating water. 8 mL of each NC dispersion with a solid content 1 mg mL^−1^ was prepared in PBS buffer (pH 7.4). 4 mL of these NC dispersions were taken in a 20 mL glass vial and 10 units of proteinase K dissolved in 100 µL PBS was added to it. For the control sample without the enzyme, 100 µL PBS was added. These solutions were shaken using a thermoshaker at 25 °C at 300 rpm. 400 µL of aliquots were taken at 0, 2, 6, 24, 48, and 72 h and filtered using Amicon ultra‐0.5 centrifugal filter unit 50 KDa (Merck Millipore) to retrieve the released cargo. Fluorescence was measured using a microplate reader (Infinite M1000 Tecan, Switzerland) with *λ*_ex_ = 546 nm and *λ*_em_ = 568 nm. The fluorescence of the 0 h aliquot without filtration was taken as a 100% release value.

### Encapsulation of Adjuvants R848 and diABZI

Adjuvant encapsulated protein NC was synthesized following the protocol described above for the synthesis of protein NCs, where the adjuvants dissolved in DMSO were added to the dispersed phase before mixing it with the continuous phase. A detailed description is given in the Supporting Information.

### Isolation of Dendritic Cells from Bone Marrow

Dendritic cells were generated from the bone marrow of 10‐to19‐weeks‐old C57BL/6 mice as per the protocol described by Bros et al.^[^ ^25^ ^]^ Briefly, bone marrow cells were collected by flushing the femur and tibia of the mouse with a culture medium described by Passlick et al..^[^ ^13^ ^]^ The cells were cultured in Petri dishes (Ø 94 mm; Greiner Bio‐One) with the same medium. On day 3, 5 mL of the same medium was added to each dish, and on day 6, half of the culture medium was replaced with fresh medium. On days 7–9, non‐adherent immature BMDCs (iDCs) were harvested and seeded on 24‐well suspension culture plates (2 × 10^5^ cells mL^−1^; Greiner Bio‐One, Frickenhausen, Germany) or 96‐well suspension culture plates (2 × 10^5^ cells mL^−1^; Greiner Bio‐One, Frickenhausen, Germany) for DC maturation and cytokine secretion experiments, respectively. For the DC‐based uptake assay, the BMDCs (2 × 10^5^ cells mL^−1^) were seeded on 12‐well suspension culture plates (Greiner Bio‐One, Frickenhausen, Germany). On day 3, 500 µL of medium were added, and then half of the medium was replaced with fresh medium on day 6. On days 7–9, free adjuvants, LPS, and protein NC were added to the BMDCs for 24 h. Cytokine secretion and DC maturation were analyzed via flow cytometry.

### Flow Cytometry Analysis

Cells were harvested and washed (1% FBS in PBS), then Fc receptors were blocked by incubating with rat anti‐mouse CD16/CD32 Ab (clone 2.4G2) for 10 min at 4 °C to prevent unspecific binding. Afterward, samples were stained with monoclonal antibodies against mouse cell surface markers, CD80‐PE (clone 16‐10A) (eBioscience), CD86‐FITC (clone GL1) (BD biosciences), CD11c‐PE‐Cy‐7 (clone N418) (Thermo Fisher Scientific) for 30 min at 4 °C. To determine cellular viability, a fixable viability dye (FVD) (eF1506) was added, which enables the identification of dead cells. BMDC left untreated served as an internal control. Sample measurements were performed using an Attune NxT (Thermo Fisher) flow cytometer. MFI data for cell maturation markers were obtained using defined gating strategies and analyzed with Attune NxT software v3.2.1 (Thermo Fisher). The percentage of maturation markers shown in the supporting information (Figures S24 and S25, Supporting Information) was determined using FlowJo software v10.8.1 (Ashland, OR, USA).

### Cytokines Secretion

The amount of TNF‐α, IL‐1β, IL‐12p70, and IFN‐β in the culture supernatants was measured by LEGEND plex MU anti‐virus response panel (13‐plex, Biolegend). Stained beads were analyzed by Attune NxT (Thermo Fisher) flow cytometer and LEGENDplex software (Biolegend).

### Stability Experiment

Stability experiments were performed 4 weeks after the synthesis of the protein NCs. To evaluate any potential adjuvants released from the protein NC, supernatants were separated from protein NC formulations via Amicon Centrifuge filter 50 KDa and centrifuged at 4000 g for 10 min at 4 °C. Subsequently, BMDCs were treated with varying concentrations of protein NCs or to the equivalent volumes of their respective supernatants for 24 h. Then secretion of cytokines was analyzed using the methods, as described previously.

### Protein Corona Preparation

The protein corona (PC) preparation was performed according to a standard procedure as described by the group earlier with slight adaptations.^[^ ^14^, ^26^ ^]^ Briefly, an aliquot of the NCs and the PS‐NPs (unfunctionalized) accounting for a common total surface area of 0.1 m^2^ was incubated with 1 mL of human citrate plasma (cP) for 1 h at 37 °C and shaking (300 rpm). cP only without nanocarrier addition was subjected to the PC preparation workflow in parallel as a negative control to illustrate the enriched protein background due to the preparation process. After the incubation, PS NP was centrifuged for 1 h 20,000 g as described before,^[^ ^14b^ ^]^ and the protein NCs were centrifuged at 1000 g for 1 h in order to preserve NC stability. The supernatant with excess of proteins was removed and the nanocarrier pellets were redispersed in 1 mL PBS and centrifuged for another 1 h with the same conditions as mentioned above to remove non‐bound and loosely bound proteins. After the centrifugation, the supernatant was discarded and this washing procedure was performed another two times with PBS. After the last washing step and supernatant removal, the nanocarrier pellets were redispersed in 100 µL of desorption buffer (69.35 mm SDS, 62.5 mm Tris‐HCl) and incubated at 95 °C for 5 min, shaking (300 rpm) to desorb remaining hard corona proteins. In order to collect the corona proteins and to separate them from the nanocarrier, the dispersions were centrifuged a last time, the supernatant was collected and further used for protein quantification, SDS‐PAGE, and LC‐MS analysis. The protein concentrations were determined with the Pierce 660 nm Protein Assay Reagent with the usage of the Ionic Detergent Compatibility Reagent (both Thermo Scientific, Germany) due to the SDS presence, according to the manufacturer's instructions. A standard calibration curve was created with a bovine serum albumin solution (Sigma‐Aldrich, Germany). Using an Infinite M1000 plate reader (Tecan, Switzerland), the absorption was measured at 660 nm.

### SDS‐PAGE and Silver Staining

For a qualitative control of the protein corona pattern on an SDS‐PAGE gel, 2 µg were diluted of protein with demineralized water to a total volume of 26 µL and added 4 µL of NuPAGE sample reducing agent and 10 µL of NuPAGE LDS sample buffer (both Invitrogen/Thermo Fisher, Germany). For the nanocapsule standards, 25 ng of protein were applied. The samples were subsequently incubated at 70 °C for 10 min. The total sample volume was loaded on a Bolt 10% Bis‐Tris Plus gel and electrophoresis was allowed for 1 h 20 min at 100 V in NuPAGE MES SDS running buffer (both Invitrogen/Thermo Fisher, Germany). 5 µL of SeeBlue Plus2 Pre‐Stained Standard (Invitrogen/Thermo Fisher, Germany) was co‐migrated on the gel as molecular weight marker. Protein bands were visualized with silver staining using the SilverQuest Silver Staining Kit (Invitrogen/Thermo Fisher, Germany) according to the manufacturer's instructions. Using a View Pix 1100 scanning system (Biostep, Germany), the gels were scanned.

### In‐Solution Tryptic Digestion

An in‐solution tryptic digestion prior to LC‐MS analysis was carried out as already described before with some adaptations.^[^ ^14^, ^26^, ^27^ ^]^ Samples containing SDS were loaded on Pierce Detergent Removal Spin Columns (Thermo Scientific, Germany) to remove SDS (which would otherwise affect the ionization in LC‐MS) following the manufacturer's instructions. 12 µg of proteins were precipitated using the ProteoExtract Protein Precipitation Kit (CalBioChem, Germany), following the manufacturer's instructions. Subsequently, precipitated proteins were centrifuged at 10,000 g for 10 min at room temperature and washed two times using the kit's washing solution. After removing the supernatant carefully, the protein pellet was allowed to dry at room temperature for 2 min. Subsequently, 100 µL 0.1% RapiGest SF surfactant (Waters Corporation, Germany), dissolved in 50 mm ammonium carbonate buffer, were added to the pellets. The tubes were briefly vortexed to solubilize the pellets and finally were incubated at 80 °C for 15 min shaking (300 rpm). In order to reduce disulfide bonds, 100 mm dithiothreitol (Sigma‐Aldrich, Germany) solution, prepared in 50 mm ammonium carbonate buffer, was added to the solubilized proteins to receive an end concentration of 5 mm. The reaction was allowed for 45 min at 56 °C shaking (300 rpm). A 500 mm iodoacetamide (Sigma‐Aldrich, Germany) solution, prepared in 50 mm ammonium carbonate buffer, was added to receive a final concentration of 15 mm for thiol alkylation that was performed at room temperature for 1 h in the dark. Subsequently, in a mass ratio of 50:1 (protein: trypsin), a trypsin solution (Promega, Germany) was added to the samples, and the following tryptic digestion was allowed for 17 h at 37 °C, shaking (300 rpm). The reaction was stopped by lowering the pH with the addition of 2 µL 37% hydrochloric acid and subsequent incubation for 45 min at 37 °C. Finally, the samples were centrifuged at 13 000 g for 15 min at 4 °C to separate aggregates. The supernatant containing the peptides was used for LC‐MS analysis.

### Liquid Chromatography Coupled to Mass Spectrometry (LC‐MS)

For absolute quantification, peptide samples were prepared as described previously and spiked with 50 fmol µL −1 Hi3 E.coli Standard (Waters Corporation, Germany). The sample volume was adjusted with LC‐MS grade water (Merck, Germany) containing 0.1% formic acid (Sigma‐Aldrich, Germany).^[^ ^28^ ^]^ The LC‐MS unit was operated with a nanoACQUITY UPLC system coupled to a Synapt G2Si mass spectrometer (both Waters Corporation, Germany). The UPLC was run with a C18 nanoACQUITY trap column (5 µm, 180 µm × 20 mm) and a C18 analytical reversed‐phase column (1.7 µm, 75 µm × 150 mm; both Waters Corporation, Germany), applying a gradient of 2% to 37% of mobile phase B over 70 min, with (A) 0.1% v/v formic acid in water and (B) 0.1% v/v formic acid in acetonitrile (Biosolve, Germany) as solvents. The samples were injected with a flow rate of 0.3 µL min^−1^ and the reference components Glu‐Fibrinopeptide and LeuEnkephalin (both Sigma‐Aldrich, Germany) with a flow rate of 0.5 µL min^−1^. Injected samples were ionized by electrospray ionization (ESI) and the NanoLockSpray source was set to positive mode. All measurements were carried out as technical triplicates and with settings as described earlier in the group using the software MassLynx 4.1 (Waters Corporation): Resolution mode and data‐independent acquisition (MSE) with a mass to charge range of 50–2,000 Da, scan time of 1 s, ramped trap collision energy from 20 to 40 V, and data acquisition of 90 min.

### Protein Identification from Protein Corona

MassLynx measurement files (see previous section) were imported into Progenesis QI 2.0 for Proteomics (Nonlinear Dynamics) to process and analyze the proteomic data according to an earlier described procedure.^[^ ^29^ ^]^ In brief, the noise reduction threshold for low energy was set as 120 counts, elevated energy as 25 counts, and peptide intensity as 750 counts. The human proteome database with reviewed proteins and the sequences for Bos taurus hemoglobin (P01966, P02070, P02081, P06642, P06643) were retrieved from UniProt (swiss prot) and imported as fasta file into Progenesis as a reference for protein identification. To allow absolute quantification, the protein sequence for the standard protein, Hi3 E.coli standard, was added to the reference data. The following settings were applied for the identification: one missed cleavage, maximum protein mass of 600 kDa, fixed carbamidomethyl modification for cysteine, variable oxidation for methionine, a minimum of three assigned fragments per peptide, a minimum of two assigned peptides per protein, a minimum of five assigned fragments per protein, and a score parameter below 4.^[^ ^14b^ ^]^

### Statistical Analysis

Data are expressed as mean ± SD of the values. ANOVA was applied for multiple groups’ comparison using GraphPad Prism 9.01 (GraphPad Software, La Jolla, USA). The protein concentration measurements in the Pierce assay were performed in technical duplicates. The data are presented as mean values and standard deviations. For LC‐MS analysis the samples were measured in technical triplicate. The heat map data present relative protein amount as the mean values for % protein based on all identified proteins. Supporting Information lists with all identified proteins include mean values and standard deviations.

## Conflict of Interest

The authors declare no conflict of interest.

## Supporting information

Supporting Information

Supporting Information

## Data Availability

The data that support the findings of this study are available from the corresponding author upon reasonable request.
