# Supplementary material for: Interfacial Denaturation at the Droplet Simplifies the Formation of Drug‐Loaded Protein Nanocapsules to Enhance Immune Response of Cells
Source: Adv Sci (Weinh). 2024 Jul 8;11(34):2403668. doi: 10.1002/advs.202403668 (PMC11425835; doi:10.1002/advs.202403668)
Supplement: Supplementary file 1 — Supporting Information [file ADVS-11-2403668-s001.pdf]

ADVANCED SCIENCE
Open Access

Supporting Information

for *Adv. Sci.*, DOI 10.1002/advs.202403668

Interfacial Denaturation at the Droplet Simplifies the Formation of Drug-Loaded Protein Nanocapsules to Enhance Immune Response of Cells

Sharafudheen Pottanam Chali, Jinhong Kang, Michael Fichter, Kai Robert Speth, Volker Mailänder and Katharina Landfester**

Supplementary Information

Interfacial denaturation at the droplet simplifies the formation of drug-loaded protein nanocapsules to enhance immune response of cells

Sharafudheen Pottanam Chali,[a,+] Jinhong Kang,[a,b,+] Michael Fichter,[a,b] Kai Robert Speth,[a] Volker Mailänder,[a,b,*] Katharina Landfester[a,*]

[a]Max Planck Institute for Polymer Research, Ackermannweg 10, 55128 Mainz, Germany

[b]University Medical Center Mainz, Langenbeckstraße 1, 55131 Mainz, Germany

[+] These authors contributed equally

* Corresponding authors

Table of Contents

1.1 Materials 4

1.2 Encapsulation of adjuvants in Hb-NC 4

1.3 Encapsulation of adjuvants in Hb-OVA-NC.......... 5

1.4 Encapsulation of adjuvants in Ker-NC 6

1.5 Encapsulation of adjuvants in Ker-OVA-NC 7

1.6 Supplementary figures and tables 8

Figure S 1: Synthesis of protein nanocapsules (NC) by miniemulsification method 8

Figure S 2: SEM images of Hb-NC synthesized at A,C) 25 ℃ and B,D) 40 ℃ 9

Figure S 3: SEM images of Hb-NC synthesized at 60 ℃.......... 9

Figure S 4: TEM images of Hb-NC synthesized at 60 ℃ 10

Figure S 5: Encapsulation of cargoes in protein nanocapsules (NC) by miniemulsification method... 11

Figure S 6: SEM images of Ker-NC synthesized at A,D) 25 ℃, B,E) 40 ℃ and C,F) 60 ℃. 11

Figure S 7: TEM images of Ker-NC synthesized at 60 ℃. 12

Figure S 8: Circular Dichroism (CD) chromatograms of A) Hb, B) OVA measured at different temperatures. C) Percentage of α-helix and random coil obtained from CD measurements for OVA at different temperatures. 12

Figure S 9: Unfolding curves obtained from nano DSF measurements of A) keratin, B) Ker-NC and their corresponding first derivative plot C) keratin, D) Ker-NC 12

Figure S 10: Unfolding curves obtained from nano DSF measurements of A) Hb, B) Hb-NC and their corresponding first derivative plot C) Hb, D) Hb-NC 13

Figure S 11: A) Synthesis of ovalbumin nanocapsules (OVA-NC) from OVA by miniemulsification and heating. DLS chromatograms of OVA-NC in cyclohexane and water obtained at B) 40 ℃ and D) 60 ℃. SEM images of OVA-NC synthesized at C) 40 ℃ and E) 60 ℃.. 13

Figure S 12: Additional SEM images of OVA-NC synthesized at A,C) 40 ℃ and B,D) 60 ℃ 14

Figure S 13: SEM images of Hb-OVA-NC synthesized at 60 ℃.......... 14

Figure S 14: TEM images of Hb-OVA-NC synthesized at 60 ℃.......... 15

Figure S 15: A) Synthesis of Ker-OVA-NC from keratin and OVA. B) DLS of Ker-OVA-NC synthesized at 40 ℃ in cyclohexane (CH) and water (W). C) SEM image of Ker-OVA-NC synthesized at 40 ℃ and D) the corresponding size distribution from SEM. 15

Figure S 16: Additional SEM image of Ker-OVA-NC synthesized at A,C) 40 °C and B,D) 60 °C..... 16

Figure S 17: TEM images of Ker-OVA-NC synthesized at 60 °C. 16

Figure S 18: SEM images of Hb-NC synthesized at 60 °C encapsulated with A) Cy5-oligo, B) Cy5-oligo, R848, C) Cy5-oligo, diABZI and D) Cy5-oligo, R848, diABZI. Inset shows the corresponding size distribution obtained from SEM measurements. 18

Figure S 19: SEM images of Hb-OVA-NC synthesized at 60 °C encapsulated with A) Cy5-oligo, B) Cy5-oligo, R848, C) Cy5-oligo, diABZI and D) Cy5-oligo, R848, diABZI. Inset shows the corresponding size distribution obtained from SEM measurements.. 18

Figure S 20: SEM images of Ker-NC synthesized at 60 °C encapsulated with A) Cy5-oligo, B) Cy5-oligo, R848, C) Cy5-oligo, diABZI and D) Cy5-oligo, R848, diABZI. Inset shows the corresponding size distribution obtained from SEM measurements. 19

Figure S 21: SEM images of Ker-OVA-NC synthesized at 60 °C encapsulated with A) Cy5-oligo, B) Cy5-oligo, R848, C) Cy5-oligo, diABZI and D) Cy5-oligo, R848, diABZI. Inset shows the corresponding size distribution obtained from SEM measurements.. 19

Figure S 22: A) SDS-PAGE and silver staining. B) Quantification of PC amount (Pierce™ assay)... 20

Figure S 23: 4-week stability test to measure the cytokines expression of supernatants from different types of protein NC.. 21

Figure S 24: Evaluation of DC maturation markers CD80 and CD86 following stimulation with adjuvant-loaded Hb-NC and Hb-OVA-NC and their relevant soluble form.. 22

Figure S 25: Evaluation of DC maturation markers CD80 and CD86 following stimulation with adjuvant-loaded Ker-NC and Ker-OVA-NC and their relevant soluble form. 23

Table S5: Table showing the characterization of adjuvant encapsulated NC………………………..17

1.1 Materials

Hemoglobin (from bovine blood) as lyophilised powder, Albumin (from chicken egg white, ≥98% purity) as lyophilised powder and Proteinase K from tritirachium album (≥ 30 units/mg) was purchased from Sigma Aldrich. Keratin (partially sulfonated, 5% in Water) was purchased from TCI. Cyclohexane (CH) (HPLC grade) was purchased from VWR. The block copolymer poly((ethylene-co-butylene)-b-(ethylene oxide)) P((E/B)-b-EO) which was used as the oil soluble surfactant was synthesized as described in literature[*Macromolecules* 34.13 (2001): 4302-4304] and consists of a poly((ethylene-co-butylene) block (NMR: Mn = 3900 g/mol) and a poly(ethylene oxide) block (NMR: Mn = 2700 g/mol). The anionic surfactant sodium dodecyl sulfate (SDS) was purchased from Sigma Aldrich. Cy5-Oligo (sequence: CCACUCCUUUCCAGAAAACU, Modifications: 5'→Cyanine 5 [Cy5]) was purchased from Eurofins. The adjuvants resiquimod (R848) and diABZI was purchased from Invivogen. Amicon Ultra centrifugal filter devices were purchased from Merck Millipore (nominal molecular weight limit (NMWL) = 50 kDa).
Human blood was provided by healthy donors at the Department of Transfusion Medicine Mainz in accordance with the Declaration of Helsinki. Citrate was added to prevent blood clotting. Human citrate plasma (cP) of ten donors was pooled and stored at -80 °C. The study was approved by the local ethics committee "Landesärztekammer Rheinland-Pfalz" (Bearbeitungsnummer: 837.439.12 (8540-F)). After thawing, cP was cleaned by centrifugation to remove protein aggregates for 30 min at 20000 g.

1.2 Encapsulation of adjuvants in Hb-NC

Table S 1: Composition of different components for the synthesis of adjuvant encapsulated Hb-NC

Sample	Hb-NC-Cy5 oligo	Hb-NC-Cy5 oligo-R848	Hb-NC-Cy5 oligo -diABZI	Hb-NC-Cy5 oligo -R848-diABZI
Hb	50 mg	50 mg	50 mg	50 mg
Water	450 µL	380 µL	370 µL	300 µL
NaCl	7.2 mg	7.2 mg	7.2 mg	7.2 mg
R848	-	0.7 mg (70µL 10mg/ml in DMSO)	-	0.7 mg (70µL 10mg/ml in DMSO)
diABZI	-	-	0.8 mg (80µL 10mg/ml in DMSO)	0.8 mg (80µL 10mg/ml in DMSO)
Cy5-oligo (136 µM)	50 µL	50 µL	50 µL	50 µL
P((E/B)-b-EO)	35.7 mg+10.7 mg	35.7 mg+10.7 mg	35.7 mg+10.7 mg	35.7 mg+10.7 mg
CH	7.5 g + 5 g	7.5 g + 5 g	7.5 g + 5 g	7.5 g + 5 g

The continuous phase was prepared by dissolving 35.7 mg P((E/B)-b-EO) in 7.5 g cyclohexane by sonication at 40 ℃ in a 40 mL vial and the dispersed phase was prepared by dissolving 50 mg Hb, 7.2 mg NaCl in water (see the Table S 1) using thermoshaker (500 rpm, 20 ℃) and Cy5-oligo in water and the adjuvants in DMSO (see the Table S 1) were added dropwise with stirring at 300 rpm. Continuous phase was added to the dispersed phase and stirred at 750 rpm for 10 minutes and then ultra-sonicated for 180 s (20 s ultrasonication, 10 s pause) with water cooling at 70% amplitude using a Branson 450W sonifier and a 1/2' tip. After the sonication, 10.7 mg P((E/B)-b-EO) in 5 g cyclohexane was added to the miniemulsion dropwise at 25 ℃ and then stirred at 25 ℃ or 40 ℃ or 60 ℃ for 18 h. Hb-NC in cyclohexane was purified using centrifugation. 8 mL of the NC dispersion was taken in two 15 mL centrifuge tubes and centrifuged at 1500 g for 30 min at 20 °C. After centrifugation, the supernatant (supernatant 1) was transferred to another set of 15 mL centrifuge tubes and the pellet was redispersed after adding 8 mL cyclohexane by shaking the tubes for 1 min in a sonication bath. The centrifugation step was repeated at 1500 g for 30 min at 20 °C. To increase the yield, supernatant 1 was centrifuged at

3000 g for 30 min at 20 °C. After centrifugation, the supernatant was disposed and the centrifugation was repeated after redispersing in 8 mL cyclohexane. Hb-NC from all the centrifuge tubes were redispersed in cyclohexane by pipetting to make a final volume of 1 mL. To transfer Hb-NC from cyclohexane to water, 500 µL of purified NC dispersion in cyclohexane was added dropwise with stirring to 5 mL SDS solution (0.1 wt%) in a 40 mL glass vial at 750 rpm and then vigorously shaken in circle motions in an ultrasound bath (25 kHz) for 5 min. Afterwards, the vial was completely covered with aluminium foil and few holes were punched into the foil with a needle at the glass opening and the dispersion was stirred for 18 h at room temperature at 750 rpm to evaporate cyclohexane. Before purification in water, the dispersion in water was shaken in the ultrasonication bath in circle motions for 1 min, then transferred to a 40 mL Amicon Centrifuge filter 50 KDa and centrifuged at 800 g for 30 min at 20 °C. Supernatant was removed and the dispersion was redispersed in 5 mL water. The centrifugation step was repeated three times and the purified NC dispersion (~2 mL) was transferred to a 4 mL glass vial and stored at 4 °C.

1.3 Encapsulation of adjuvants in Hb-OVA-NC

Table S 2: Composition of different components for the synthesis of adjuvant encapsulated Hb-OVA-NC

Sample	Hb-OVA-NC-Cy5 oligo	Hb-OVA-NC-Cy5 oligo - R848	Hb-OVA-NC-Cy5 oligo-diABZI	Hb-OVA-NC-Cy5 oligo -R848-diABZI
Hb	25 mg	25 mg	25 mg	25 mg
OVA	25 mg	25 mg	25 mg	25 mg
Water	450 µL	380 µL	370 µL	300 µL
NaCl	7.2 mg	7.2 mg	7.2 mg	7.2 mg
R848	-	0.7 mg (70µL 10mg/ml in DMSO)	-	0.7 mg (70µL 10mg/ml in DMSO)
diABZI	-	-	0.8 mg (80µL 10mg/ml in DMSO)	0.8 mg (80µL 10mg/ml in DMSO)
Cy5-oligo (136 µM)	50 µL	50 µL	50 µL	50 µL
P((E/B)-b-EO)	35.7 mg+10.7 mg	35.7 mg+10.7 mg	35.7 mg+10.7 mg	35.7 mg+10.7 mg
CH	7.5 g + 5 g	7.5 g + 5 g	7.5 g + 5 g	7.5 g + 5 g

Adjuvant encapsulated Hb-OVA-NC was synthesised following the procedure in Section 1.2. The preparation of dispersed phase was slightly modified. 25 mg Hb, 25 mg OVA, 7.2 mg NaCl was dissolved in water (see the Table S 2) using thermoshaker (500 rpm, 20 °C). Cy5 oligo in water and the adjuvants in DMSO (see the Table S 2) were added dropwise with stirring at 300 rpm.

1.4 Encapsulation of adjuvants in Ker-NC

Table S 3: Composition of different components for the synthesis of adjuvant encapsulated Ker-NC.

Sample	Ker-NC-Cy5 oligo	Ker-NC-Cy5 oligo-R848	Ker-NC -Cy5 oligo -diABZI	Ker-NC -Cy5 oligo -R848-diABZI
Keratin (partially sulfonated, 5% in Water)	500 mg (i.e. ~25 mg keratin)	500 mg (i.e. ~25 mg keratin)	500 mg (i.e. ~25 mg keratin)	500 mg (i.e. ~25 mg keratin)
NaCl	7.2 mg	7.2 mg	7.2 mg	7.2 mg
Water	35 µL	40 µL	35 µL	-
DMSO	40 µL	-	-	-
R848	-	0.35 mg (35µL 10mg/ml in DMSO)	-	0.35 mg (35µL 10mg/ml in DMSO)
diABZI	-	-	0.4 mg (40µL 10mg/ml in DMSO)	0.4 mg (40µL 10mg/ml in DMSO)
Cy5-oligo (136 µM)	50 µL	50 µL	50 µL	50 µL
P((E/B)-b-EO)	35.7 mg+10.7 mg	35.7 mg+10.7 mg	35.7 mg+10.7 mg	35.7 mg+10.7 mg
CH	7.5 g + 5 g	7.5 g + 5 g	7.5 g + 5 g	7.5 g + 5 g

Adjuvant encapsulated Ker-NC was synthesised following the procedure in Section 1.2. The preparation of dispersed phase was slightly modified. 7.2 mg NaCl was dissolved in keratin solution by stirring at 300 rpm. Additional water and/or DMSO, Cy5 oligo in water and the adjuvants in DMSO were added dropwise with stirring at 300 rpm (see the Table S 3).

1.5 Encapsulation of adjuvants in Ker-OVA-NC

Table S 4: Composition of different components for the synthesis of adjuvant encapsulated Ker-OVA-NC.

Sample	Ker-OVA-NC - Cy5 oligo	Ker-OVA-NC-Cy5 oligo-R848	Ker-OVA-NC - Cy5 oligo - diABZI	Ker-OVA-NC - Cy5 oligo -R848-diABZI
Keratin (partially sulfonated, 5% in Water)	400 mg (i.e. ~20 mg keratin)	400 mg (i.e. ~20 mg keratin)	400 mg (i.e. ~20 mg keratin)	400 mg (i.e. ~20 mg keratin)
OVA	20 mg	20 mg	20 mg	20 mg
NaCl	7.2 mg	7.2 mg	7.2 mg	7.2 mg
Water	70 µL	64 µL	56 µL	-
DMSO	50 µL	-	-	-
R848	-	0.56 mg (56µL 10mg/ml in DMSO)	-	0.56 mg (56µL 10mg/ml in DMSO)
diABZI	-	-	0.64 mg (64µL 10mg/ml in DMSO)	0.64 mg (64µL 10mg/ml in DMSO)
Cy5-oligo (136 µM)	50 µL	50 µL	50 µL	50 µL
P((E/B)-b-EO)	35.7 mg+10.7 mg	35.7 mg+10.7 mg	35.7 mg+10.7 mg	35.7 mg+10.7 mg
CH	7.5 g + 5 g	7.5 g + 5 g	7.5 g + 5 g	7.5 g + 5 g

Adjuvant encapsulated Ker-OVA-NC was synthesised following the procedure in Section 1.2. The preparation of dispersed phase is slightly modified. 7.2 mg NaCl, 20 mg OVA was dissolved in keratin solution by stirring at 300 rpm. Additional water and/or DMSO, Cy5 oligo in water and the adjuvants in DMSO was added dropwise with stirring at 300 rpm (see the Table S 4).

1.6 Supplementary figures and tables

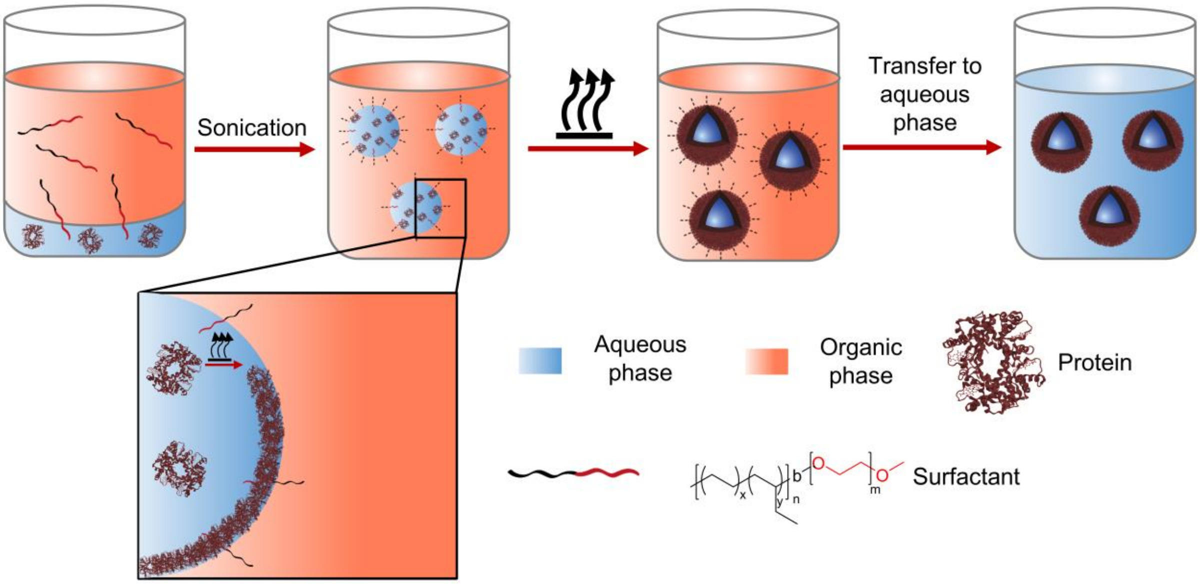

Figure S 1: Synthesis of protein nanocapsules (NC) by miniemulsification method. Proteins dissolved in the dispersed phase (water) and polymeric surfactant dissolved in the continuous organic phase were emulsified using ultrasonication. The emulsion was heated to obtain protein NCs in the organic phase. In this step, proteins get confined and denatured at the oil water interface. After purification to remove excess of the surfactant, protein NCs in cyclohexane were transferred to water using sodium dodecyl sulfate (SDS) as a surfactant, cyclohexane was evaporated at room temperature to obtain protein NCs exclusively in water and then SDS was removed by centrifugal filtration.

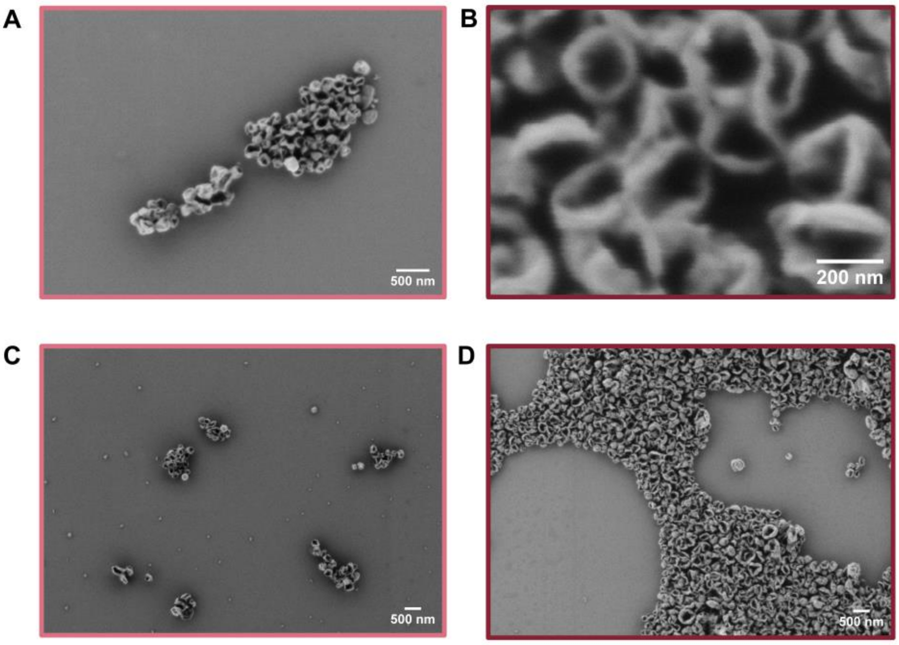

Figure S 2: SEM images of Hb-NC synthesized at A,C) 25 °C and B,D) 40 °C

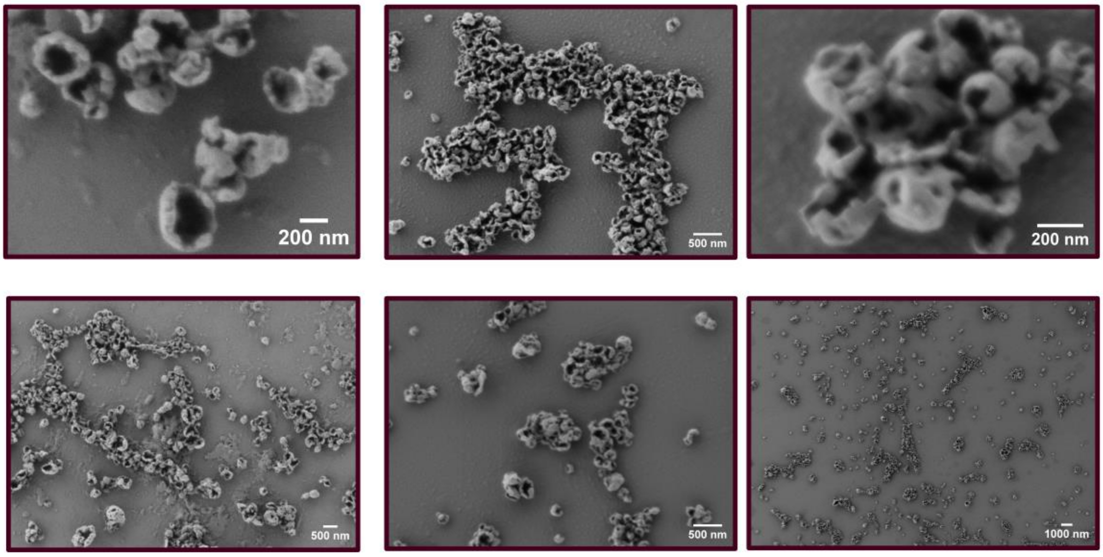

Figure S 3: SEM images of Hb-NC synthesized at 60 °C

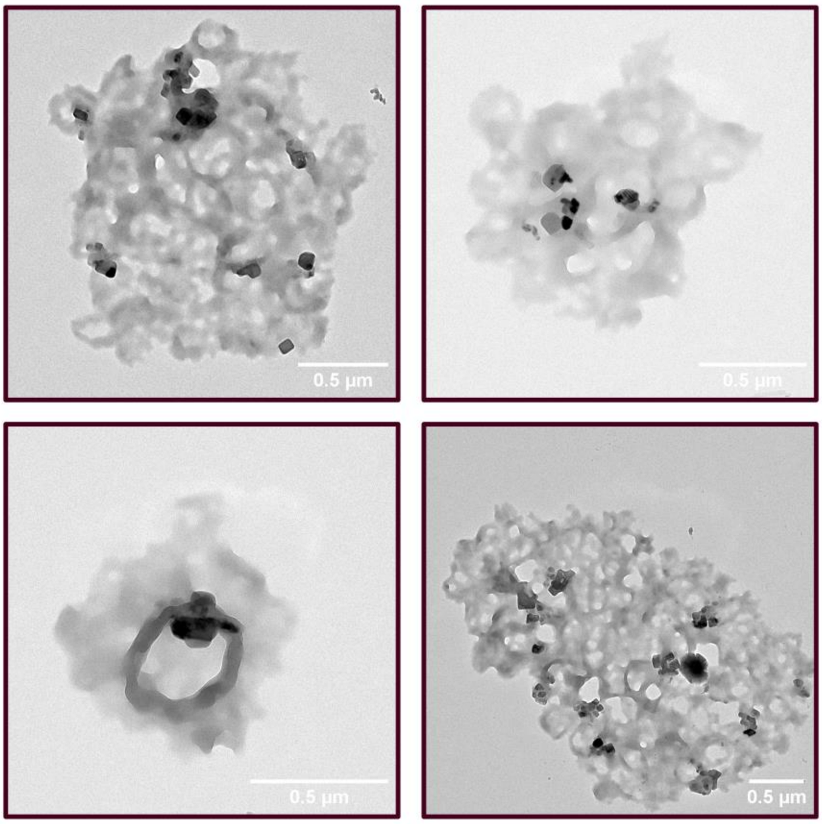

Figure S 4: TEM images of Hb-NC synthesized at 60 °C

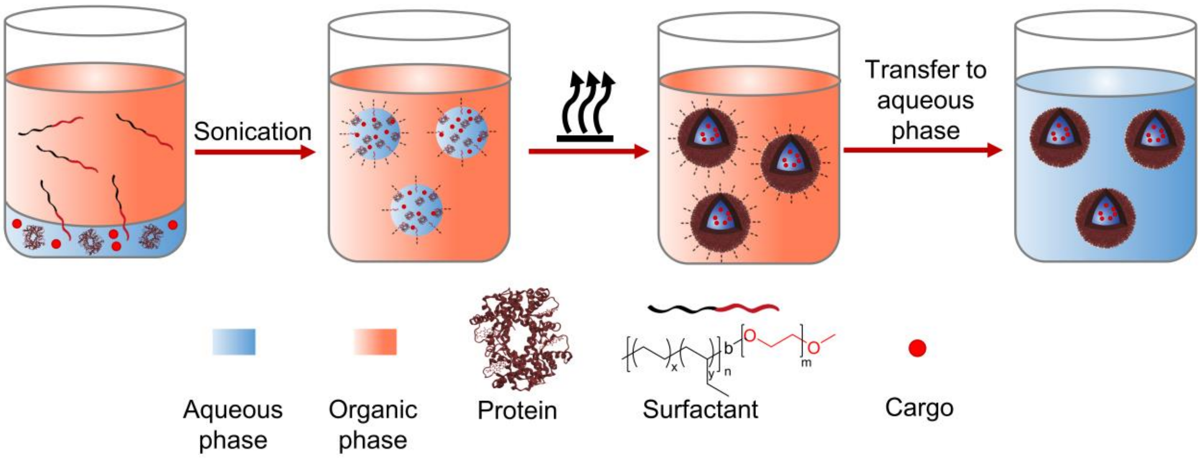

Figure S 5: Encapsulation of cargoes in protein nanocapsules (NC) by miniemulsification method. Proteins and the cargo dissolved in the dispersed phase (water) and surfactant P((E/B)-b-EO) (low HLB surfactant) dissolved in the continues phase (cyclohexane) was emulsified using ultrasonication. The emulsion was heated to obtain protein NC in the organic phase. In this step, protein get confined and denatured at the oil water interface. After purification to remove excess of the surfactant, protein NC in cyclohexane was transferred to water using SDS as a surfactant (high HLB surfactant), cyclohexane was evaporated at room temperature to obtain protein NC exclusively in water and then SDS and non-encapsulated cargoes were removed by centrifugal filtration.

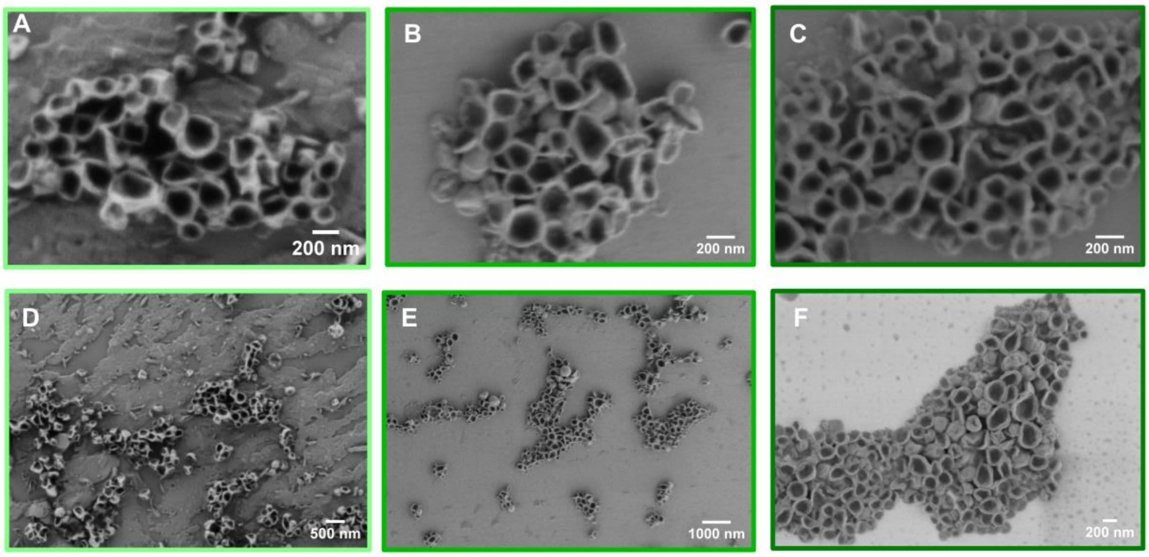

Figure S 6: SEM images of Ker-NC synthesized at A,D) 25 °C, B,E) 40 °C and C,F) 60 °C.

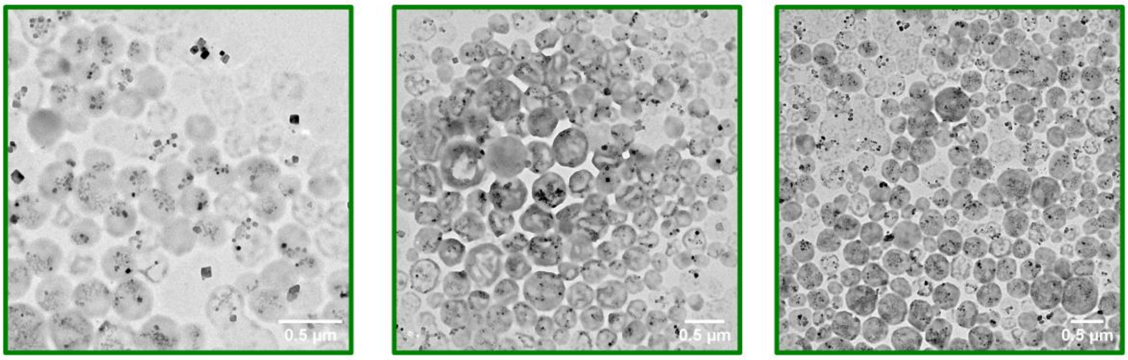

Figure S 7: TEM images of Ker-NC synthesized at 60 °C.

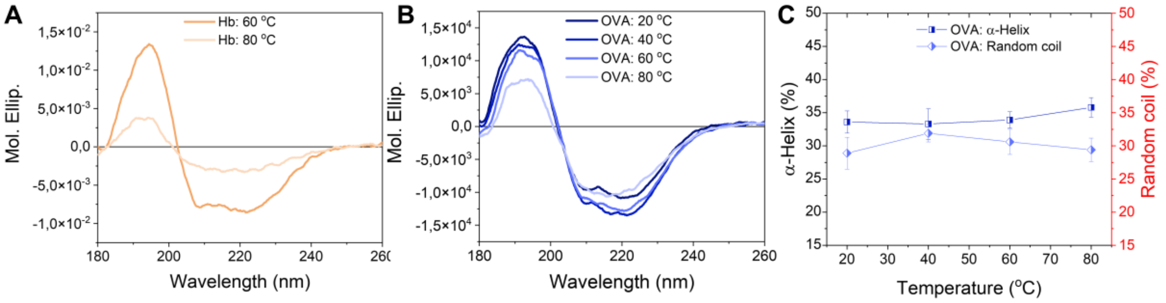

Figure S 8: Circular Dichroism (CD) chromatograms of A) Hb, B) OVA measured at different temperatures. C) Percentage of α-helix and random coil obtained from CD measurements for OVA at different temperatures.

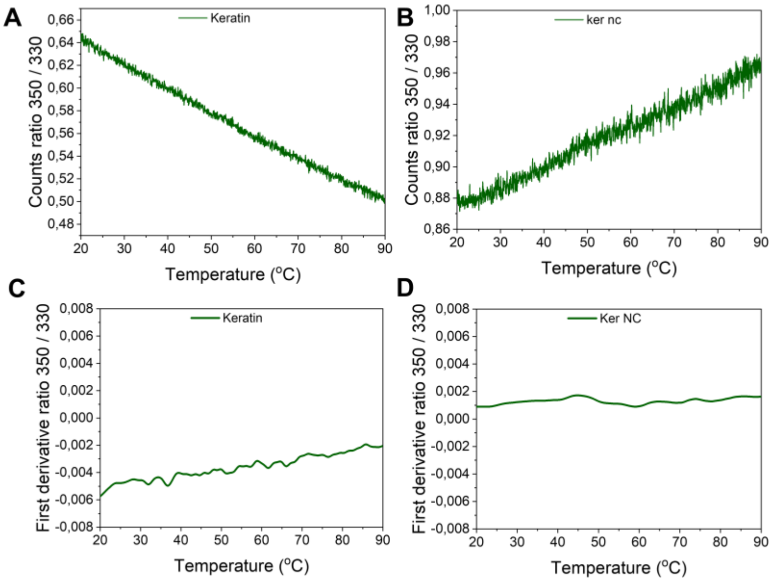

Figure S 9: Unfolding curves obtained from nano DSF measurements of A) keratin, B) Ker-NC and their corresponding first derivative plot C) keratin, D) Ker-NC

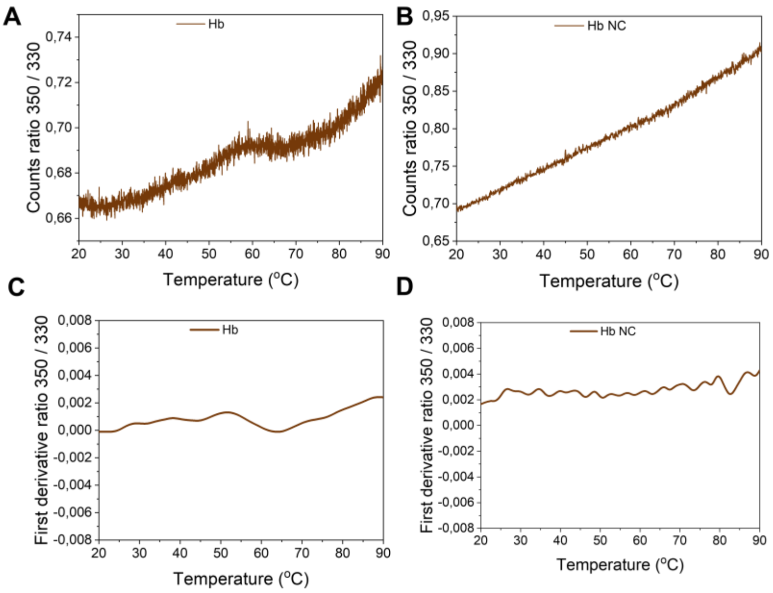

Figure S 10: Unfolding curves obtained from nano DSF measurements of A) Hb, B) Hb-NC and their corresponding first derivative plot C) Hb, D) Hb-NC

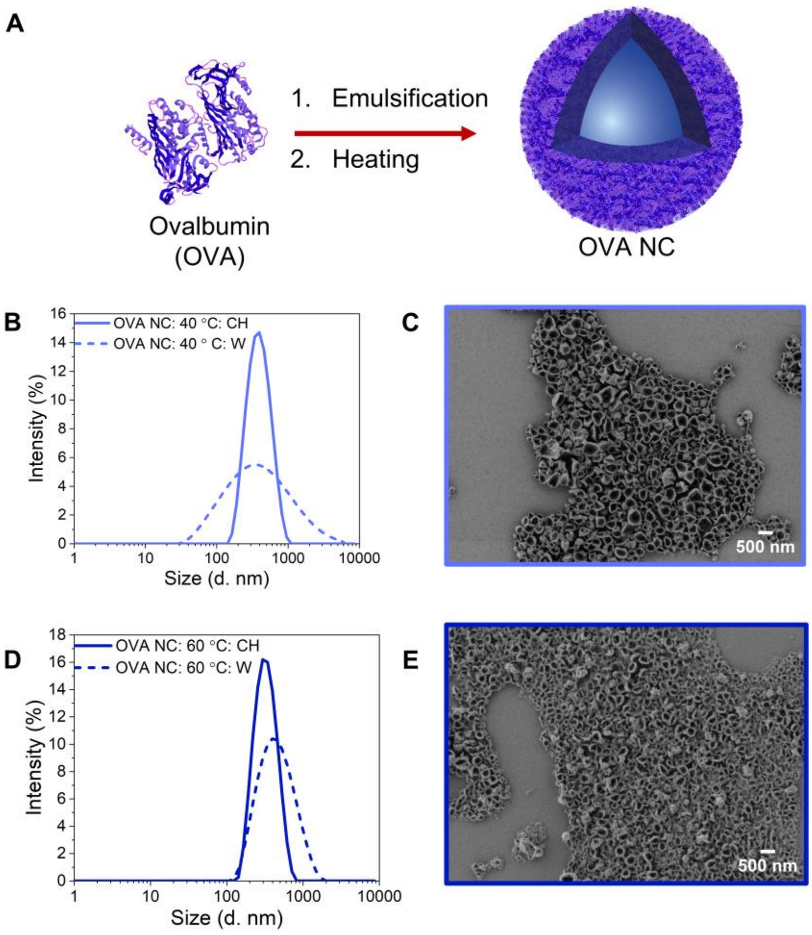

Figure S 11: A) Synthesis of ovalbumin nanocapsules (OVA-NC) from OVA by miniemulsification and heating. DLS chromatograms of OVA-NC in cyclohexane and water obtained at B) 40 °C and D) 60 °C. SEM images of OVA-NC synthesized at C) 40 °C and E) 60 °C. CH (cyclohexane), W (water).

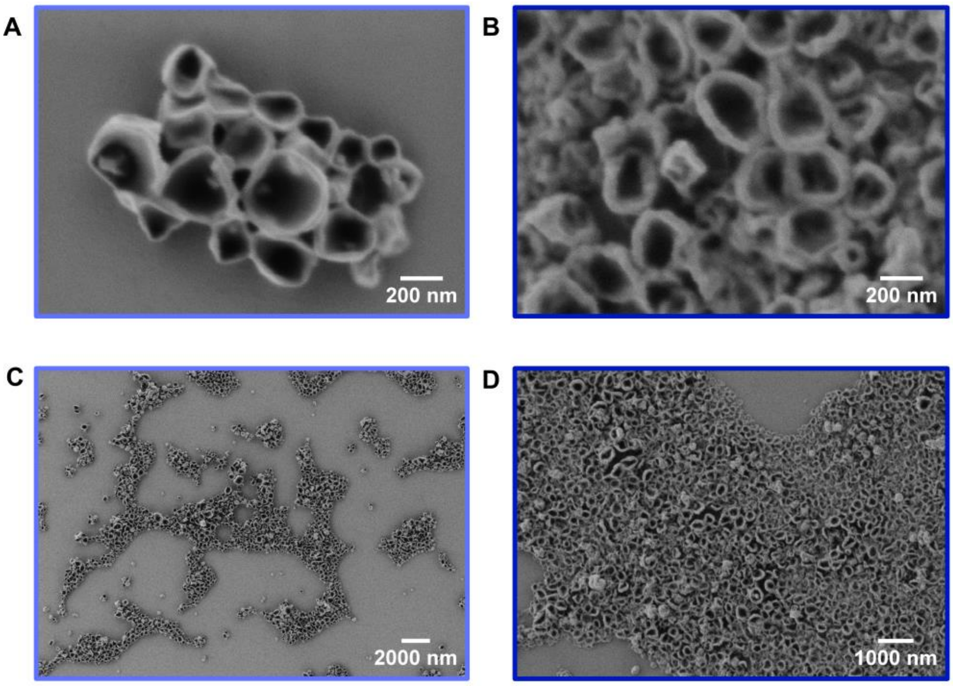

Figure S 12: Additional SEM images of OVA-NC synthesized at A,C) 40 °C and B,D) 60 °C

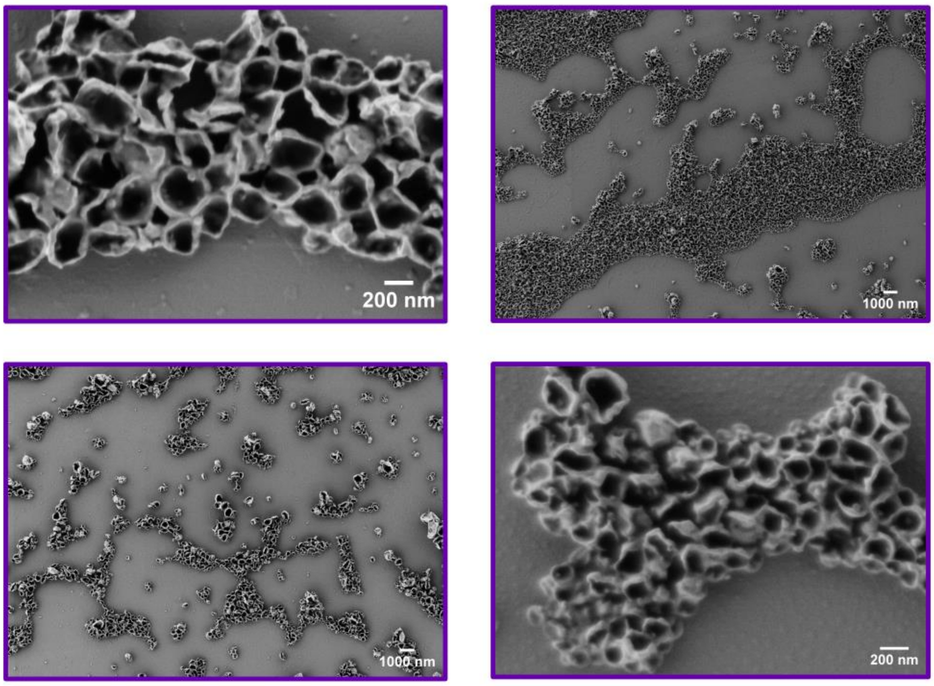

Figure S 13: SEM images of Hb-OVA-NC synthesized at 60 °C

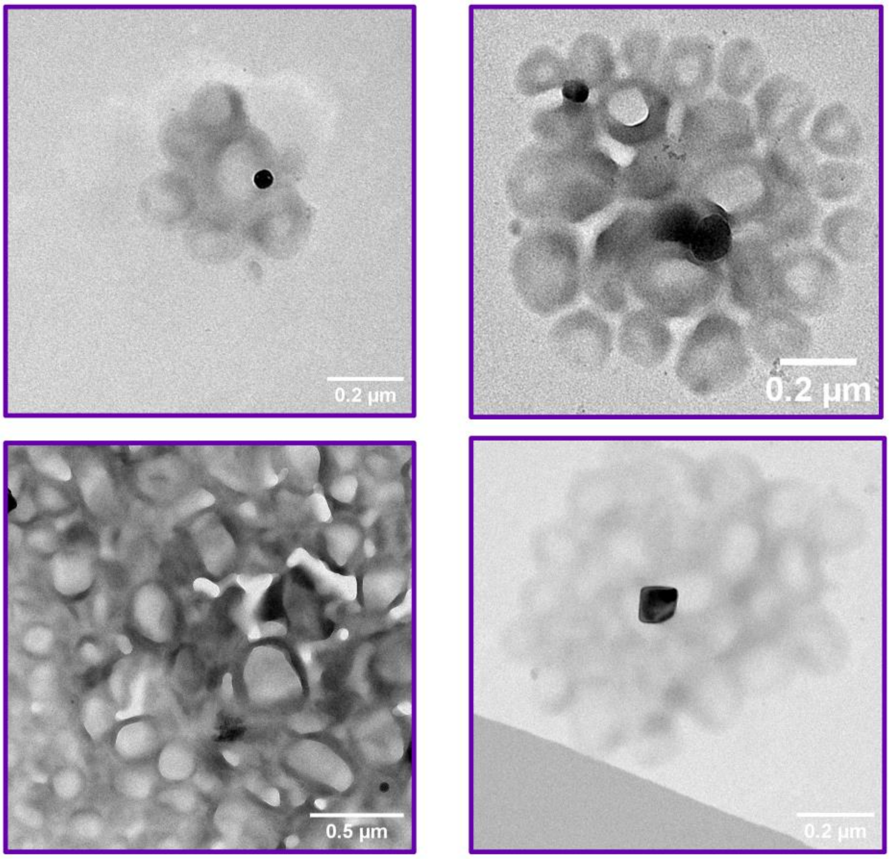

Figure S 14: TEM images of Hb-OVA-NC synthesized at 60 °C

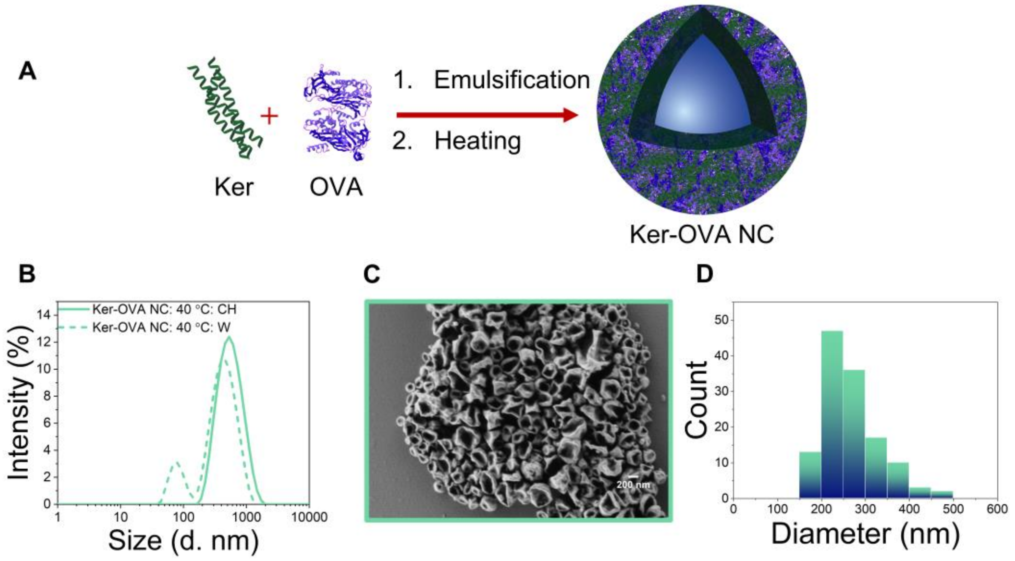

Figure S 15: A) Synthesis of Ker-OVA-NC from keratin and OVA. B) DLS of Ker-OVA-NC synthesized at 40 °C in cyclohexane (CH) and water (W). C) SEM image of Ker-OVA-NC synthesized at 40 °C and D) the corresponding size distribution from SEM.

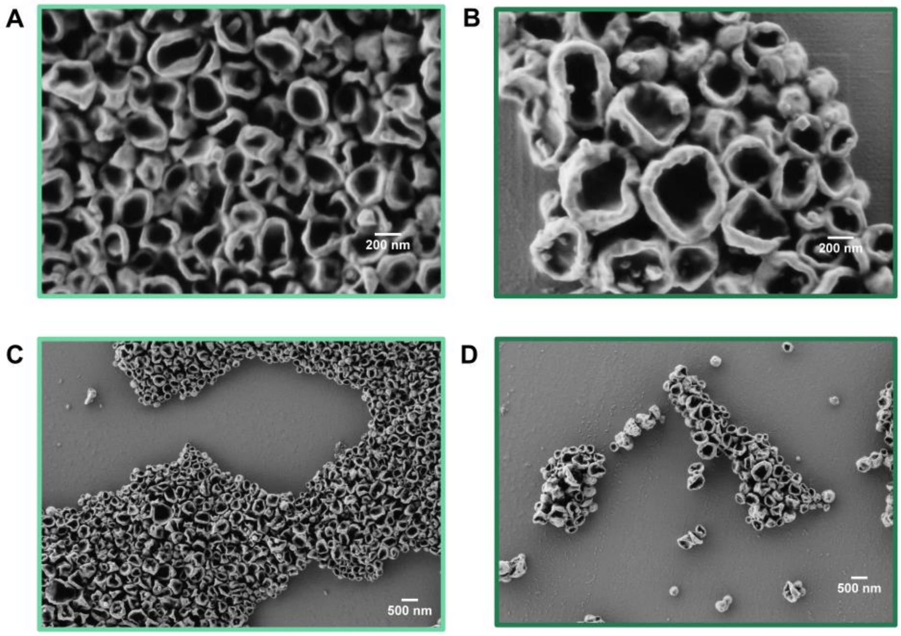

Figure S 16: Additional SEM image of Ker-OVA-NC synthesized at A,C) 40 °C and B,D) 60 °C.

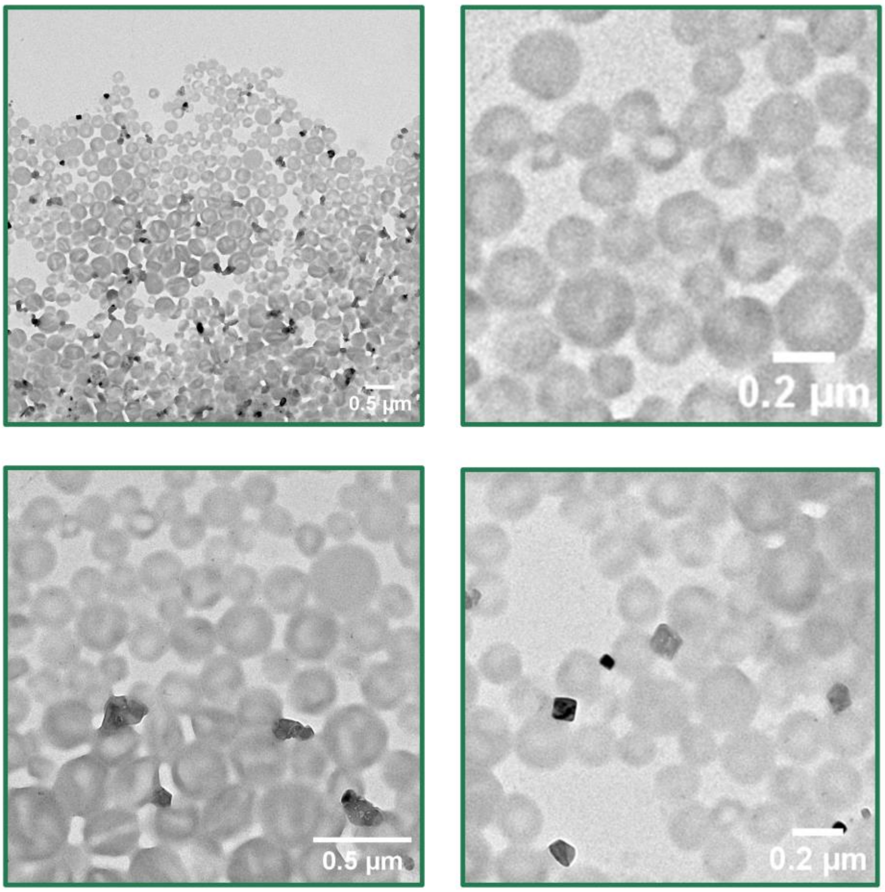

Figure S 17: TEM images of Ker-OVA-NC synthesized at 60 °C.

Table S 5: Table showing the characterization of adjuvant encapsulated NC.

Type of NC	Cargo [EE (%)]			DLS (CH)		SEM		DLS (W)		ζ (mV)
	Cy5-oligo	R848	diABZI	Diameter (nm)	PDI	Diameter (nm)	SD	Diameter (nm)	PDI	
Hb	77			310	0.1	200	37	212	0.20	-11.8
Hb	75	45		267	0.12	157	31	157	0.35	-21.8
Hb	80		85	289	0.07	178	30	167	0.26	-16.7
Hb	71	43	79	269	0.14	176	48	199	0.25	-18.5
Hb-OVA	65			257	0.07	179	33	243	0.32	-8.1
Hb-OVA	64	47		354	0.10	273	58	236	0.35	-9.1
Hb-OVA	66		95	325	0.09	232	52	220	0.35	-7.5
Hb-OVA	71	49	81	278	0.17	150	23	256	0.29	-8.5
Ker	84			329	0.10	304	56	185	0.26	-8.3
Ker	79	49		309	0.09	273	49	192	0.27	-8.8
Ker	76		81	300	0.12	254	42	195	0.28	-7.7
Ker	77	51	92	323	0.06	279	45	198	0.27	-6.9
Ker-OVA	75			371	0.14	337	54	232	0.41	-8.8
Ker-OVA	77	55		359	0.11	335	66	293	0.42	-10.7
Ker-OVA	85		88	364	0.17	325	53	290	0.45	-9.6
Ker-OVA	83	54	89	350	0.10	354	63	293	0.41	-8.8

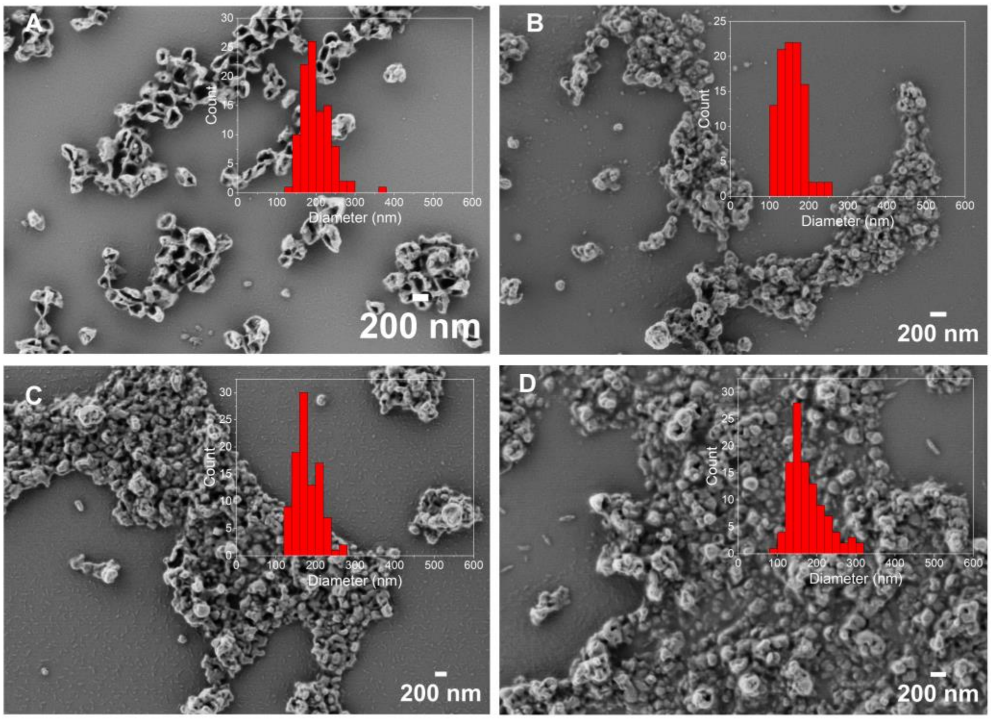

Figure S 18: SEM images of Hb-NC synthesized at 60 °C encapsulated with A) Cy5-oligo, B) Cy5-oligo, R848, C) Cy5-oligo, diABZI and D) Cy5-oligo, R848, diABZI. Inset shows the corresponding size distribution obtained from SEM measurements.

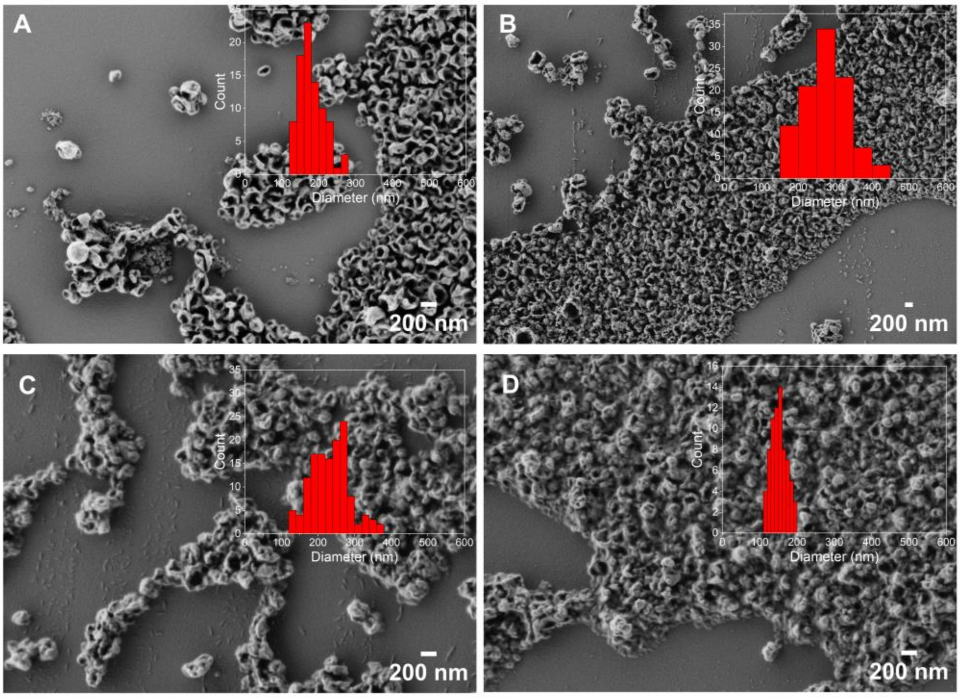

Figure S 19: SEM images of Hb-OVA-NC synthesized at 60 °C encapsulated with A) Cy5-oligo, B) Cy5-oligo, R848, C) Cy5-oligo, diABZI and D) Cy5-oligo, R848, diABZI. Inset shows the corresponding size distribution obtained from SEM measurements.

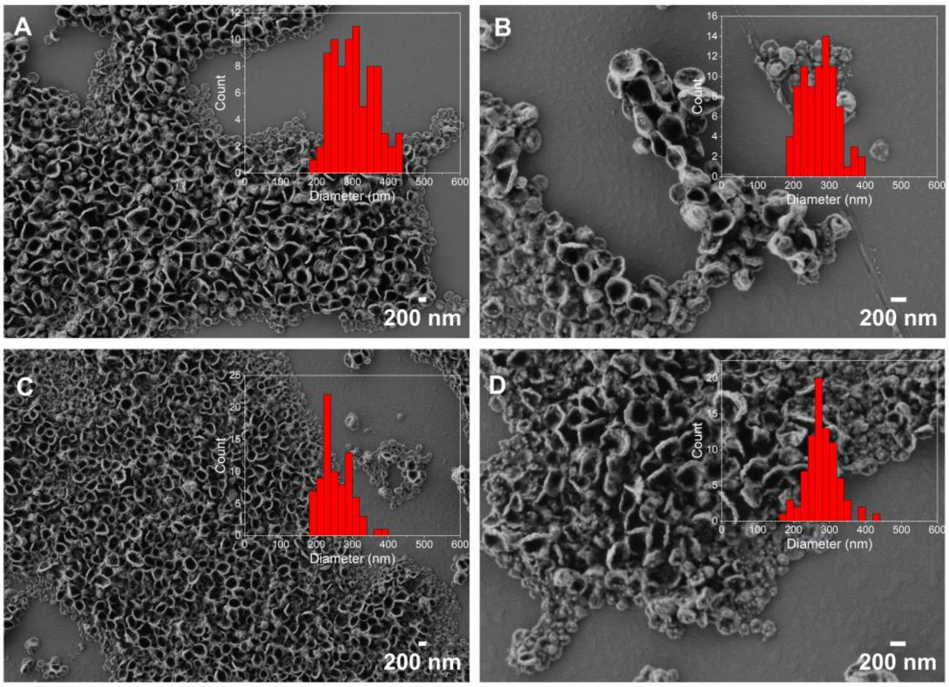

Figure S 20: SEM images of Ker-NC synthesized at 60 °C encapsulated with A) Cy5-oligo, B) Cy5-oligo, R848, C) Cy5-oligo, diABZI and D) Cy5-oligo, R848, diABZI. Inset shows the corresponding size distribution obtained from SEM measurements.

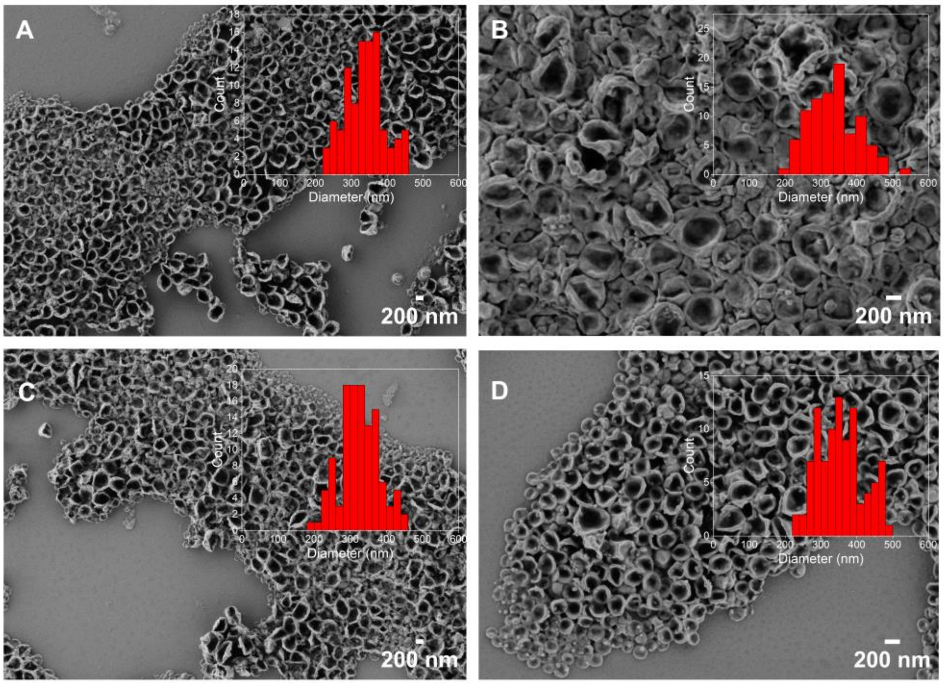

Figure S 21: SEM images of Ker-OVA-NC synthesized at 60 °C encapsulated with A) Cy5-oligo, B) Cy5-oligo, R848, C) Cy5-oligo, diABZI and D) Cy5-oligo, R848, diABZI. Inset shows the corresponding size distribution obtained from SEM measurements.

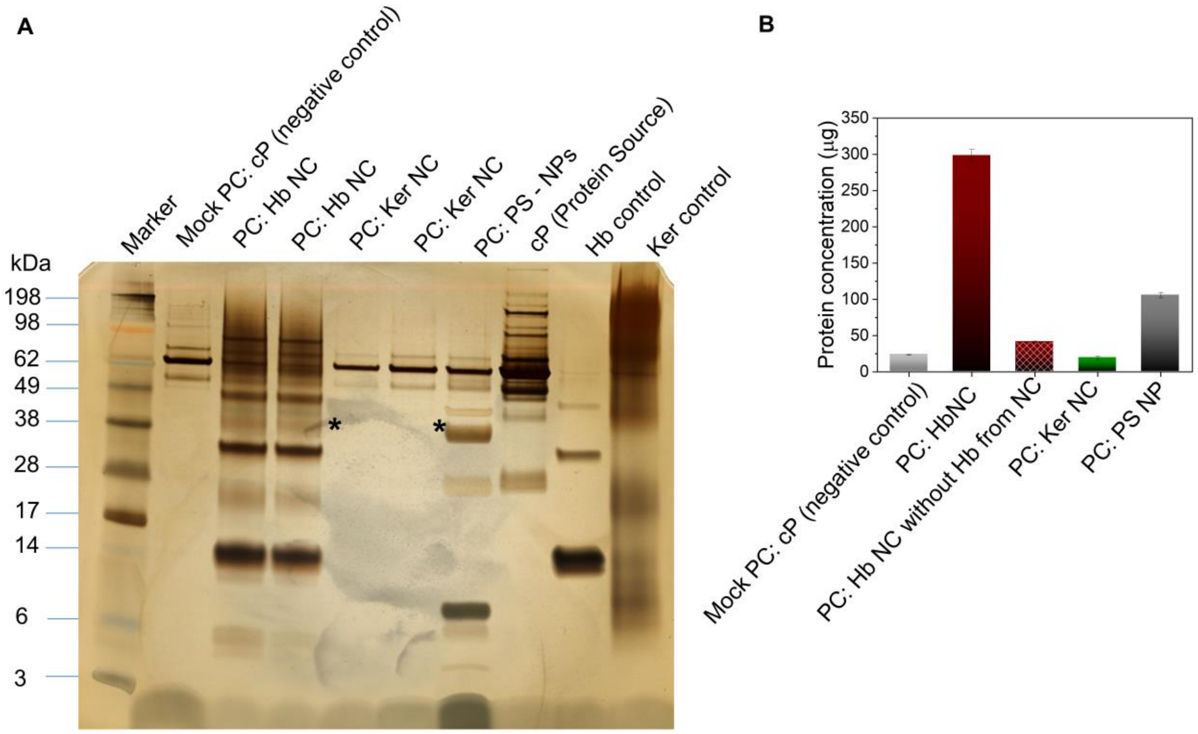

Figure S 22: A) SDS-PAGE and silver staining. Prior to LC-MS analysis, protein corona (PC) samples derived from Hb-NC, Ker-NC and PS NP were analyzed qualitatively on a gel and compared to co-migrated protein background from PC preparation process (Mock PC: cP (negative control)), pure citrate human plasma (cP (Protein Source)) and hemoglobin and keratin starting material as used for capsule synthesis (Hb and Ker controls). For Ker-NC the PC preparation process seem to be not working as no protein bands different from Mock PC sample were detected. For Hb-NC the protein pattern is more divers and different from the Hb control lane, indicating a putative PC formation. The prominent band above 38 kDa (asterisk) for the PS control might refer to clusterin as a well-known constituent of the PC of PS NP,[1] that also might appear for Hb-NC additionally. B) Quantification of PC amount (Pierce™ assay). After desorption of corona proteins with 2 % (w/v) SDS and a final centrifugation step to remove remaining Hb-NC, Ker-NC and PS NP, the desorbed proteins in the supernatant were quantified with Pierce™ 660 nm protein assay. Protein contamination by Hb-NC material itself (86.2 %) was considered from LC-MS analysis to only illustrate PC only amount (Hb-NC without Hb from NC). Mock PC preparation of sole citrate human plasma (cP) with no nanocapsules/particles served as a negative control (cP negativ control) and illustrate protein background due to the PC preparation process.

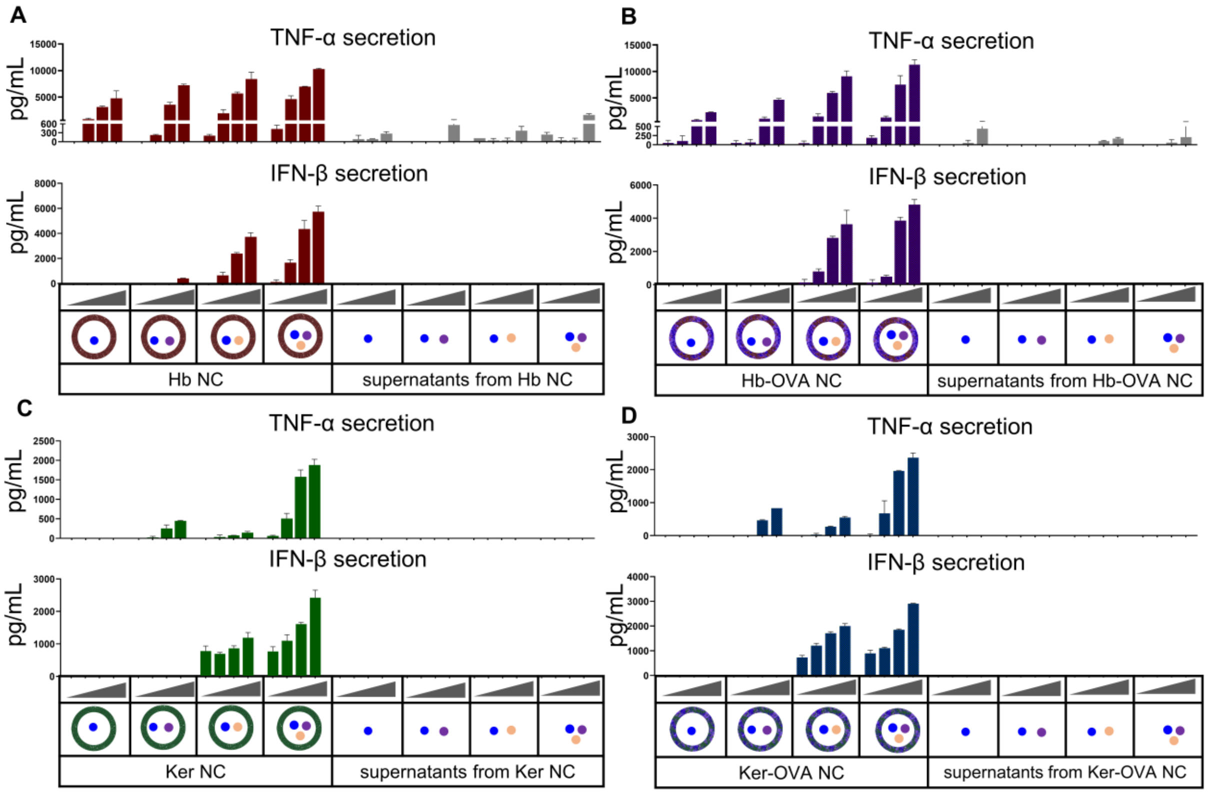

Figure S 23: 4-week stability test to measure the cytokines expression of supernatants from different types of protein NC. BMDCs were treated with varying concentrations of protein NC (1, 3, 10, 30 µg/mL) or with the corresponding volumes of their supernatants for 24 h. (A) Hb-NC (B) Hb-OVA-NC (C) Ker-NC (D) Ker-OVA-NC. Mean ± SD, n=2.

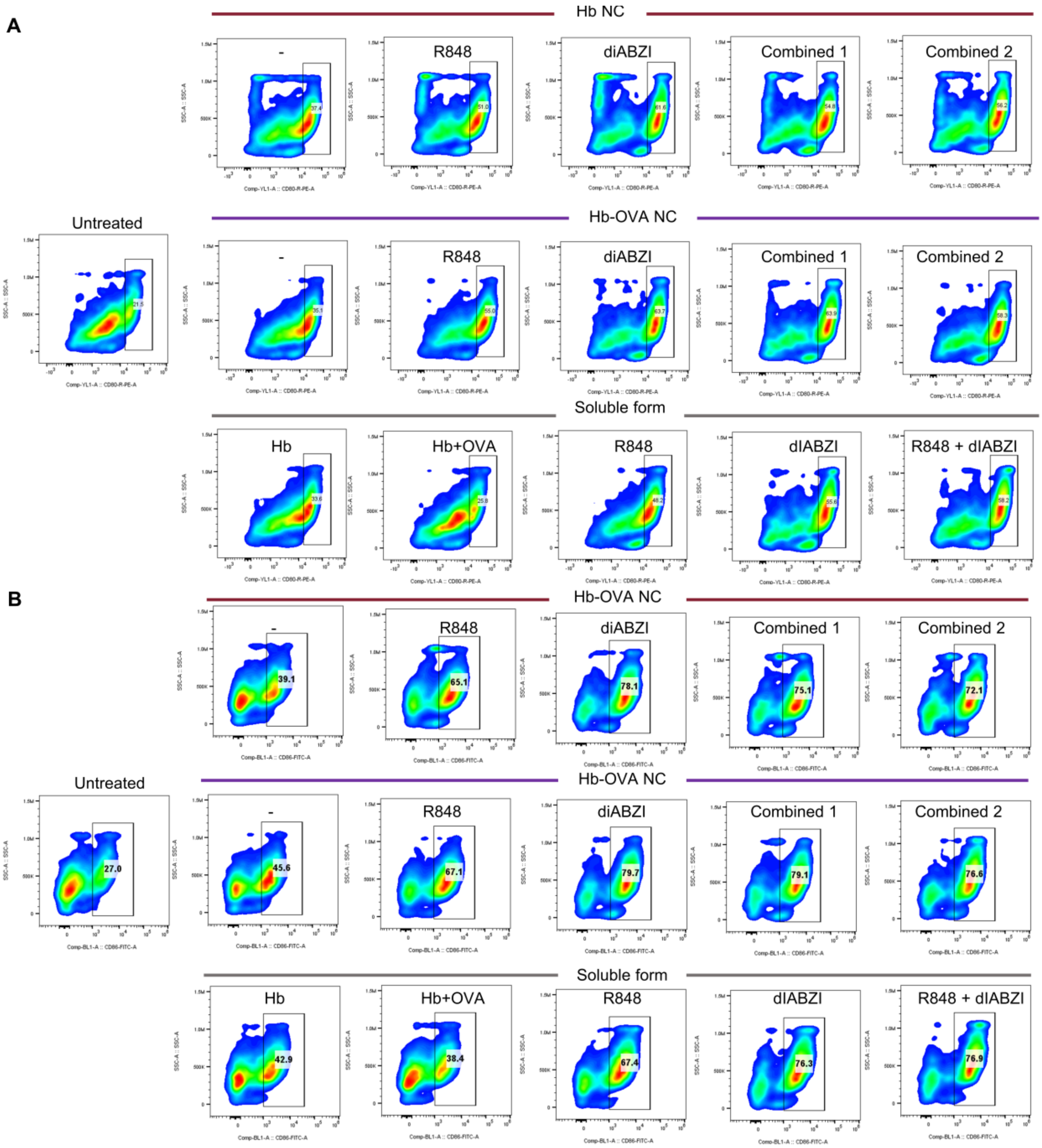

Figure S 24: Evaluation of DC maturation markers CD80 and CD86 following stimulation with adjuvant-loaded Hb-NC and Hb-OVA-NC and their relevant soluble form. *In vitro* stimulated CD11c^{+} BMDC were subjected to flow cytometry analysis to determine the levels of CD80 and CD86 expression. The shown graphs are representative of three independent experiments each. (A) CD 80 expression (B) CD 86 expression. Combined 1 indicates adjuvants encapsulated together in NC and combined 2 indicates adjuvants are encapsulated separately in NC and then the NC are combined.

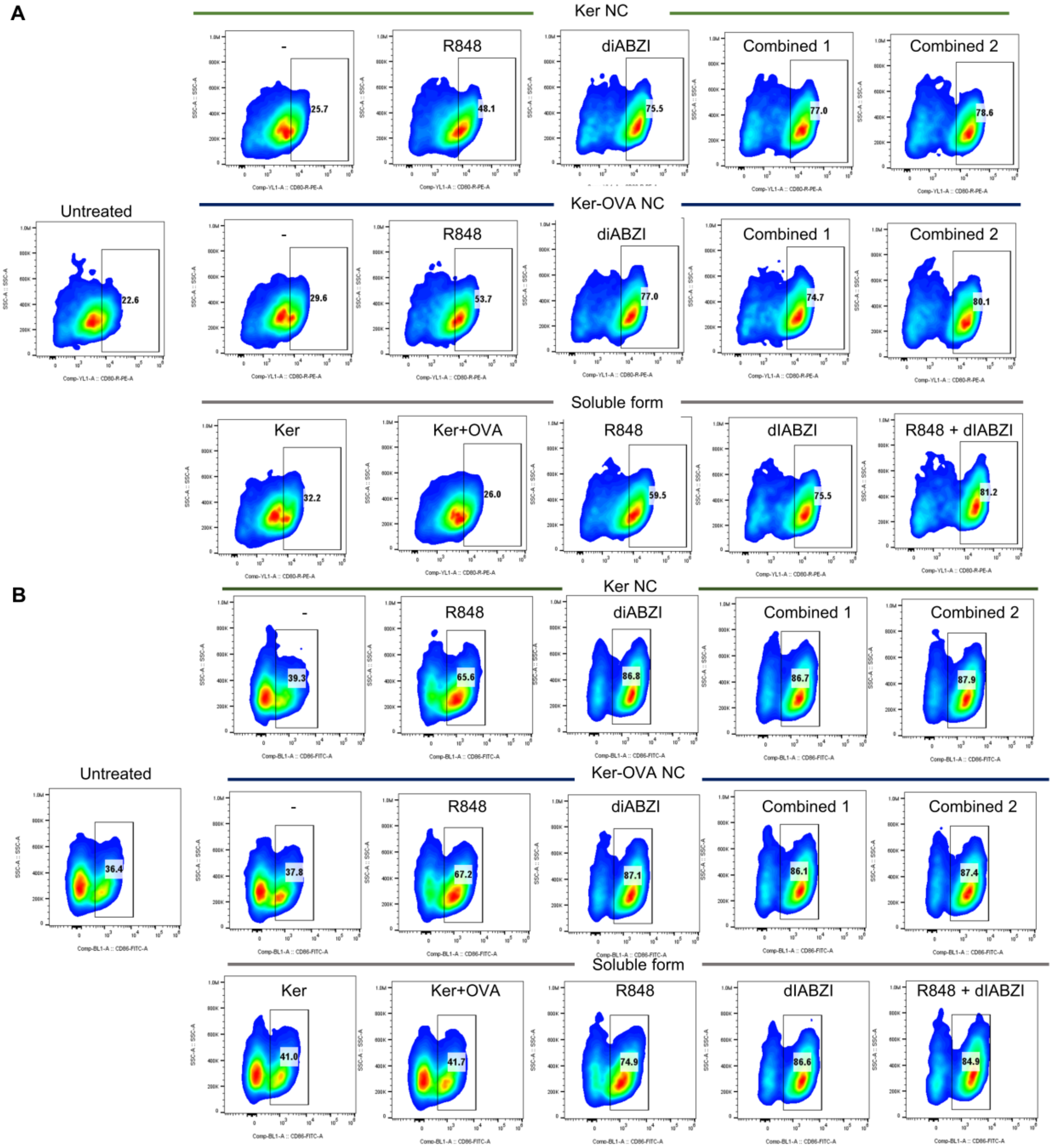

Figure S 25: Evaluation of DC maturation markers CD80 and CD86 following stimulation with adjuvant-loaded Ker-NC and Ker-OVA-NC and their relevant soluble form. *In vitro* stimulated CD11c^{+} BMDC were subjected to flow cytometry analysis to determine the levels of CD80 and CD86 expression. The shown graphs are representative of three independent experiments each. (A) CD 80 expression (B) CD 86 expression. Combined 1 indicates adjuvants encapsulated together in NC and combined 2 indicates adjuvants are encapsulated separately in NC and then the NC are combined.

1 Kokkinopoulou, M., Simon, J., Landfester, K., Mailänder, V. & Lieberwirth, I. Visualization of the protein corona: towards a biomolecular understanding of nanoparticle-cell-interactions. *Nanoscale* **9**, 8858-8870 (2017).
